# Planning intensive care unit admission after elective major abdominal surgery: good clinical practice document by SIAARTI-SIC-ANIARTI

**DOI:** 10.1186/s44158-025-00239-w

**Published:** 2025-04-14

**Authors:** Bruna Lavezzo, Giandomenico Biancofiore, Ersilia Luca, Roberto Balagna, Elena Bignami, Ugo Boggi, Rita Cataldo, Giuseppe Chiaramonte, Andrea Cortegiani, Umberto Fiandra, Roberta Mariani, Matteo Manici, Alessia Mattei, Liliana Sollazzi, Luigi Tritapepe, Martina Tosi, Stefano Turi, Mauro Zago, Paola Aceto

**Affiliations:** 1 Anesthesia and Intensive Care Unit, SS Annunziata Hospital, Savigliano, Azienda Sanitaria Locale Cuneo1, Cuneo, Italy; 2 Division of Transplant Anesthesia and Critical Care, Azienda Ospedaliera Universitaria Pisana, University of Pisa, Pisa, Italy; 3 Department of Emergency, Anesthesiological and Reanimation Sciences, Fondazione Policlinico Universitario Agostino Gemelli IRCCS, Rome, Italy; 4 Emergency Department Azienda Sanitaria Locale Città di Torino, Anaesthesia and Intensive Care Unit, Martini Hospital, Turin, Italy; 5 Anesthesiology, Intensive Care and Pain Medicine, University Hospital of Parma, Parma, Italy; 6 Division of General and Transplant Surgery, University of Pisa, Pisa, Italy; 7 Operative Research Unit of Anesthesia and Intensive Care, Fondazione Policlinico Universitario Campus Bio-Medico, Rome, Italy; 8 Anesthesia and Critical Care Department IRCCS, ISMETT-Istituto Mediterraneo Per I Trapianti E Terapie Ad Alta Specializzazione, Palermo, Italy; 9 Section of Anesthesia, Analgesia, Intensive Care and Emergency, Department of Surgical Oncological and Oral Science, Paolo Giaccone Polyclinic University of Palermo, Palermo, Italy; 10 Department of Quality, Risk Management and Accreditation, Azienda Ospedaliera Universitaria Città Della Salute E Della Scienza Di Torino, Turin, Italy; 11 Department of Anesthesiology, Intensive Care and Pain Treatment, University of L’Aquila, L’Aquila, Italy; 12 Department of Basic Biotechnological Science, Intensive Care and Peri-Operative Clinics, Università Cattolica del Sacro Cuore, Rome, Italy; 13 Department of Anesthesia and Intensive Care, Sapienza University of Rome, Rome, Italy; 14 Department of Anesthesia and Intensive Care, San Camillo-Forlanini Hospital, Rome, Italy; 15 Anaesthesia and Intensive Care Department, University Hospital of Modena, Modena, Italy; 16 Department of Anesthesia and Intensive Care, IRCCS San Raffaele Scientific Institute, Milan, Italy; 17 Robotic and Emergency Surgery Department, General and Emergency Surgery Division, A. Manzoni Hospital, ASST Lecco, Lecco, Italy

**Keywords:** Intensive care unit, Abdominal surgery, Planning admission, Risk stratification, Frailty

## Abstract

Postoperative complications (PCs) are a major cause of mortality following elective major abdominal surgery (EMAS). The increasing complexity of abdominal procedures, particularly in oncology, may significantly affect patient outcomes. However, this has also introduced a higher variability in postoperative management, and the use of tailored approaches to address critical issues such as hemodynamic stabilization, infection management, and respiratory failure. While elective admission to intensive care units (ICU) is a standard practice to manage high-risk surgical patients, ICU resource allocation is often influenced by local practices and bed availability.

This document presents a framework for preoperative ICU admission planning after EMAS. It focuses on the identification of patient and surgical risk factors—using established scoring systems—and provides statements to determine ICU admission. The aim is to optimize resource allocation, reduce PCs, and prevent unplanned ICU admissions. This good clinical practice statement was developed through a multidisciplinary panel formed by selected members coming from SIAARTI (Italian Society of Anesthesia Analgesia Resuscitation and Intensive Care), SIC (Italian Society of Surgery) and ANIARTI (National Association of Critical Area Nurses).

The designed scientific board developed, through a systematic literature review and a consensus methodology, a roadmap for defining the priorities of perioperative care based on the complexity of the patient and the surgical procedure. Eventually, the panel worked out statements about six voted queries that could have supported the preoperative indication to postoperative ICU admission.

Evaluation of patients’ characteristics, comorbidities, and surgical factors are all essential to plan ICU admission for immediate postoperative patient care after EMAS.

The presence and severity of comorbidities, assessed through various severity scores, play a crucial role in predicting PCs and guiding ICU admission decisions. Tools such as the American Society of Anesthesiologists physical status, Charlson Comorbidity Index, and Rockwood Frailty Index, along with surgical risk scores and intraoperative events, help define the need for intensive care. Preoperative frailty assessment—achieved using the Clinical Frailty Scale—is essential to anticipate postoperative care needs. Finally, during the postoperative phase, continuous monitoring and reassessment in the post-anesthesia care unit are key to determine whether ICU admission is required. Establishing high-dependency units and tailored care pathways based on individual patient needs and available resources will enhance patient outcomes and optimize postoperative care.

**Supplementary Information:**

The online version contains supplementary material available at 10.1186/s44158-025-00239-w.

## Background

Postoperative complications still represent one of the leading causes of post-operative mortality.

In recent years, the progressive increase in high-complexity abdominal surgery and its remarkable advances have improved the treatment of many pathologies, especially oncologic ones. However, this change has caused a great increase and variability of patients, who require different tailor-made management due to many critical issues, such as hemodynamic and fluid therapy optimization, early identification of infective complications, evaluation and management of hemorrhagic and thromboembolic risks, pain control, and the prevention and management of respiratory failure [1, 2].

Elective admission to the ICU is a standard of care to manage many surgical procedures, and it is indicated depending on the early risk of PCs. However, ICU is not always used due to local behaviours or intensive/semi-intensive care bed availability.

Generally, before surgery procedure there are four main factors leading to schedule ICU admission of post-operative patient [3]:

1. Intrinsic risk of surgical procedure, based, for example, on the American College of Surgeons National Surgical Quality Improvement Program (ACS NSQIP) Surgical Risk
2. Patient risk related to physical clinical condition (cardiovascular, renal, neurological, etc.)
3. Unpredictability of major complications during the procedure (“Crashing Risk”)
4. Support and monitoring not available outside of the ICU

The definition of major or high-risk surgery usually includes a functional status marker associated with an index of the level of complexity of the procedure.

The peri-operative risk of major surgery may be further increased in high-risk patients, who present a high probability of complications and mortality [3].

There is no clear consensus in literature on the benefit of ICU admission; moreover, the impact on mortality reduction is not easy to evaluate.

Several studies suggest that higher-intensity assistance is not even associated with a greater benefit and improved outcomes for surgical patients, thus it does not lead to an efficient use of resources after major surgeries [4].

Overestimating (overtriage) the need for ICU admission for low-risk post-operative patients does not improve outcomes, but it increases costs, whereas underestimating (undertriage) the need for high-risk postoperative patients is associated with higher morbidity and mortality risks [5].

According to scientific literature, the purpose of this proposal for good clinical practices is to evaluate which patient characteristics and available tools could be used for risk assessment and, consequently, decide the pre-surgical planning of ICU admission.

The elaborated statements aim to provide the basis for a decision-making roadmap used to establish which patients undergoing high-risk complexity abdominal surgery could benefit from a planned ICU admission. This approach seeks to reduce post-operative complications and/or unplanned admission, as well as appropriately allocate logistic and economic resources.

This roadmap cannot be used in all cases due to differences among regions and available facilities to provide different levels of peri-operative assistance with higher intensity care. These services currently represent intermediate levels of care or specifically dedicated ICUs, for specific post-operative patients (high-intensity care unit, dedicated post-operative care unit, sub-intensive care unit).

According to the panel, it is crucial to collaboratively develop peri-operative corporate roadmaps to establish care priority levels depending on the type of patient, surgical procedures, and structural resources available.

## Methods

Clinical trial number: not applicable.

### Panel composition

On August 27th, 2021 a multidisciplinary and inter-society working group (WG) was formally appointed, formed by experts selected depending on their skills in their relevant field.

This panel consists of 13 experts, selected by the board of Directors of SIAARTI, based on their clinical and scientific expertise. In the experts’ selection and appointment, national scientific societies relevant to each specialty were involved. In particular, the National Association of Critical Care Nurses “Associazione Nazionale Infermieri di Area Critica” (ANIARTI) and the Italian Society of Surgery “Società Italiana di Chirurgia” (SIC) have participated in the process.

Moreover, the work included two specialists in search strategy who conducted the systematic literature review (M.T., S.T.).

This document was then externally reviewed by one anesthesiologist-intensivist with expertise in methodology (A.C.) and two other experts (R.C., L.T.).

### Queries and definition of statements

A scientific board of experts, through extensive literature research, identified the relevant areas of interest and defined the queries that were agreed in the first phase of Delphi process. After a systematic review of literature, a list of corresponding statements was elaborated for each query. The selection and degree of consensus on selected statements was established according to a modified Delphi process, as planned according to SIAARTI regulations.

Specifically, during the first collaborative scoping workshop held virtually on October 22, 2021, the panel discussed the critical issues and queries addressed in this document.

All suggested clinical queries arising from this discussion were then rationalized and collected from the panel coordinator. All experts involved were called to give their opinions on the priority and suitability of clinical queries through an online form. Their opinions were given using an ordinal Likert scale, based on the UCLA-RAND methodology (minimum score, 1—completely disagree, maximum score, 9—completely agree).

In particular, the rating scale was divided into 3 sections:

- 1–3: rejection/disagree (not appropriate)
- 4–6: uncertain
- 7–9: agree/support (appropriate)

The consensus was considered gained when at least 75% of the experts, excluding methodologists and search specialists, assigned scores within the same interquartile range 1–3, 4–6, 7–9.

Upon discussion among the coordinator and panellists, the literature search experts applied the following search strategy restriction:

- Temporal restriction: including only the studies published from 2000
- The type of articles included were original articles, case series, case reports, narrative reviews, systematic reviews, meta-analyses, position papers, and guidelines. Articles not in English were excluded.

The results of the review, indicated in detail in Supplementary information 1: Appendix 4, were shared with all panel experts.

Subsequently, the panel was divided into multidisciplinary working subgroups.

Each subgroup was assigned two clinical queries to produce the statements and supporting rationales.

The experts shared the produced contents by each subgroup with the panel coordinator and with the SIAARTI clinical research office. In line with UCLA-RAND methodology, the complete list underwent voting, so that every expert could indicate their consensus level.

The same voting method used for this assessment was employed for evaluating each clinical query.

### Funding and disclosure

The authors did not receive any grant or financial support for this work.

The content of this document has not been funded by any entity, nor has it been influenced by any entity.

The authors declared no conflicts of interest.

Potential conflicts of interest have been analyzed and disclosed by all authors in accordance with the guidelines provided by the National Center for Clinical Excellence, Quality, and Safety of Care.

## Results

Out of the 8 evaluated clinical queries, only 7 obtained the panel’s agreement in the first round (see Supplementary information 1: Appendix 1). The remaining query, despite a reformulation proposal, did not achieve the panel’s agreement in the second round of voting (see Supplementary information 1: Appendix 2). Therefore, the panel agreed not to include this query in the document.

Furthermore, during the manuscript elaboration phase, the panel proposed to merge two clinical queries (formerly CQ6 and 7) into a single clinical query.

The queries approved by the panel were subsequently shared with the two literature search specialists, who conducted a systematic literature review using PubMed. The search and its reporting were conducted according to PRISMA principles (see Supplementary information 1: Appendix 4).

All rationales and statements achieved appropriateness (IQR 7–9) already in the first online vote round. The voting results are fully reported in Supplementary information 1: Appendix 3 and briefly reported for every query and statement (median score, IQR, agreement %).

###  Queries and concerning statements (Table 1 )

**Table 1:** Statements

**Question 1. What are the patient’s characteristics/comorbidities to objectively and systematically define the appropriateness of ICU admission?**	
1.1	The presence of comorbidities, their number, and their severity increase the risk of postoperative complications, although this is linked even to other peri-operative factors.
1.2	Each comorbidity requires a specific evaluation using available severity scores to establish the degree of organ and/or systemic function impairment, the possibilities for pre-operative optimization, and the planning of an appropriate treatment in case of post-operative worsening or decompensation.
1.3	Post-operative instability forecast, possible organ failure decompensation or worsening linked to the physiopathological effects of surgery on patients with organ impairment, even in a compensation phase, should be evaluated following the available scores. This evaluation guides the indication for the need of an immediate post-operative admission in ICU or in a higher-level care unit.
**Question 2. What are the appropriate tools for objectively and systematically defining the indication for postoperative ICU admission?**	
2.1	Based on the current literature evidence, the most appropriate strategy seems to be to plan the ICU admission need for patients depending on the presence of multiple pre-existing conditions associated with several non-modifiable pre-existing factors (e.g., ASA score ≥ 3, Charlson Comorbidity index ≥ 2, Rockwood Frailty Index ≥ 0.25, Revised Cardiac Risk Index (Lee Criteria) ≥ 2, BMI > 60, male gender, diabetes, sarcopenia) and/or in case of new or intra-operative events (e.g., Surgical APGAR score ≤ 7, intra-operative bleeding, hypotension requiring vasopressor support, respiratory issues) that require an intensive monitoring/treatment in the immediate post-surgery period
2.2	When predicting the need for ICU admission, the main factors to evaluate are the type of surgery and the patient’s comorbidities. Specific hospital organizations could contribute to this decision (e.g., availability of high dependency unit as step down ICU).
**Question 3. What are the appropriate tools to assess the degree of frailty in pre-operative patients to determine post-operative ICU admission?**	
3.1	The evaluation of frailty conditions (as a multidimensional syndrome, prevalent among elderly patients, but not exclusively age-related, and significantly associated with adverse postoperative events) should be included in the pre-operative evaluation process
3.2	Although there is no high-quality evidence supporting a specific frailty assessment tool, the Clinical Frailty Scale (CFS) seems to be the most accurate and practical tool since it is fast and user friendly. These characteristics make it preferable for a routine approach in the pre-surgical evaluation process
3.3	The combination of frailty screening tests with risk assessment scores for postoperative complications (i.e.,ACS NSQIP Surgical Risk) could provide a better estimation of the surgical risk and could guide decisions regarding post-operative monitoring in intensive or intermediate intensive environments. In elderly patients, in case of positivity to the frailty screening tests and before elective surgery that can be postponed, a geriatric multi-dimensional evaluation – which is also available in “short-form” – could be considered to identify possible peri-surgical optimization areas. Therefore, the use of of a multidisciplinary agreement in each center would be useful to establish the tools to be used for a comprehensive pre-operative evaluation in order to improve the pre-operative clinical status, whenever possible
**Question 4. What tools allow to assess the surgical impact and/or surgical technique on the single patient’s physiology in order to establish whether there is an indication for post-operative intensive/sub-intensive care unit admission?**	
4.1	The intrinsic risk associated with surgical procedures is a predicting factor for post-operative outcomes. The several pre-operative scores elaborated to estimate the surgical risk include even variables linked to patient characteristics (e.g., Apgar Surgical Score, ACS NSQIP Surgical Risk). Therefore, the impact of surgical risk cannot be separated from patient-specific characteristics
4.2	Most surgical risk scores currently available do not include the surgical approach nor a pre-operative optimization. Minimally-invasive techniques (laparoscopic, robotic-assisted) and multimodal prehabilitation (according to Enhanced Recovery After Surgery (ERAS) programs) should be encouraged, when possible, to reduce the surgical impact on frail patients and/or patients with organ dysfunctions
**Question 5. Can some criteria or assistance profiles be identified for postoperative monitoring and support in order to define intermediate assistance levels among the surgery ward and ICU?**	
5.1	For certain high-complexity surgical disciplines, it may be beneficial to establish units with higher intensity of care than standard surgical wards, such as high-dependency units where continuous monitoring of vital functions is provided. The fundamental strategies to optimize post-operative courses are patient selection and staff training
5.2	To define the level of post-operative assistance for each patient, it is necessary to consider the following: 1) age, comorbidities, and risk factors for metabolic and organ complications; 2) type, invasiveness, and duration of surgical procedure; 3) unexpected intra-operative events; 4) the clinical and metabolic patient’s conditions upon awakening and the onset of immediate postoperative complications, including POD 5) available facilities and resources (including human ones)
**Question 6. Which parameters could be reassessed at the end of surgery and before PACU discharge to confirm, indicate, or modify indication for ICU admission?**	
6.1	The clinical feature of the patient in the immediate post-operative period is closely related to pathophysiological interactions between pre-operative clinical conditions, surgery impact and anesthesia on organs and systems, mechanical ventilation effects, fluids, blood and hemoderivates administered
6.2	Mild-high-risk patient observation in PACU is crucial for post-operative management as it allows for the evaluation and monitoring of vital parameters and immediate treatment. It could represent a transitory phase for the patient’s treatment, before establishing ICU transfer, depending on neurological status, cardiorespiratory and renal functions, metabolic compensation, and possible immediate post-operative complications. Score utilization at discharge could support a comprehensive evaluation of the patient
6.3	Neurological state and/or cardiovascular/respiratory function alterations are determining factors for re-evaluating the patient's destination. The concomitant use of scores and checklists, as well as clinical judgment, can make one decide for the most appropriate intensity level of care required by the patient
6.4	Organ and/or metabolic dysfunction of moderate to high grade, even if the patient is stable, may require continuous post-operative monitoring, feasible only in high assistance level settings. For this reason, hospital Governance should encourage the development of differentiated care pathways for surgical patients, based on single missions and local resources

#### Patient characteristics and comorbidities for objectively and systematically determining ICU admission appropriateness

**Question 1. What are the patient’s characteristics/comorbidities to objectively and systematically define the appropriateness of ICU admission? **Median score. 9 (IQR 7, 25–9); agreement: 92.86%

Statement 1.1: median score: 9 (IQR 6–9); agreement: 84.61%

The presence of comorbidities, their number, and their severity increase the risk of postoperative complications, although this is linked even to other peri-operative factors.

Statement 1.2: median score: 8 (IQR 5–9); agreement: 84.61%

Each comorbidity requires a specific evaluation using available severity scores to establish the degree of organ and/or systemic function impairment, the possibilities for pre-operative optimization, and the planning of an appropriate treatment in case of post-operative worsening or decompensation.

The calculation of these scores should be systematically included in the pre-operative assessment by both the anaesthesiologist and the surgeon.

Statement 1.3: median score: 9 (IQR 7–9); agreement: 100%

Post-operative instability forecast, possible organ failure decompensation or worsening linked to the physiopathological effects of surgery on patients with organ impairment, even in a compensation phase, should be evaluated following the available scores. This evaluation guides the indication for the need of an immediate post-operative admission in ICU or in a higher-level care unit.

Rationale

The post-operative course is influenced by a complex interaction between the surgery performed, the patient’s pre-operative health status, and specific intra and post-operative events. Unplanned ICU admission immediately after surgery is often associated with worse outcomes and an increased need for organ failure support. Therefore, an appropriate triage to establish the indication for immediate post-operative ICU admission could have an impact on the post-surgical course [6, 7].

Pre-operative comorbidities are now recognized as predictors of both post-operative morbidity and mortality and should be evaluated and established through available scores or classification systems [8].

Despite significant inter-observers’ variability, the ASA classification is now widely accepted as correlating with post-operative morbidity and mortality, which is higher in patients with poor physical conditions and severe systemic diseases. However, the ASA classification is generic and not sufficient to stratify peri-operative risks, and it does not consider the potential improvement of some pre-existing conditions associated with comorbidities (such as malnutrition or anaemia) in elective surgeries [9].

Advanced biological age, which often does not correlate with chronological age, is a condition associated with multiple comorbidities [10].

In the current literature, there is a lot of evidence about elderly patients and discriminating factors such as the number of associated comorbidities, the presence of OSAS, and the corresponding ASA class for indication for post-operative ICU admission [11].

The number of comorbidities [11, 12], their severity, and the possibility of improving preoperative compensation influence the treatment strategies to be implemented immediately before surgery, such as in the case of COPD [13], or in patients suffering from arrhythmias [14]. The indication for ICU stems from advanced monitoring needs and/or unavailable treatment in ordinary surgery wards [15].

Cardiovascular and pulmonary diseases, OSAS included, [11, 13, 16] are among the most studied due to the existence of appropriate pre-operative evaluation scores, and their early identification, which allow for adequate peri-operative treatment [17].

OSAS poses a risk for difficult mask ventilation and intubation, as well as airways obstruction in the post-operative period, thus requires an appropriate monitoring. This monitoring depends not only on the type of surgery and anaesthesia, but also on other patient-related factors, such as age and BMI [18].

Moreover, several conditions reported in literature, regardless of the degree of compensation, may have an impact on post-operative outcomes.

Among these there are diabetes, which is associated with a higher risk of post-operative complications, especially when poorly controlled [19, 20], electrolyte imbalances, such as sodium disturbances associated with certain pathologies [21], anaemia, which is frequently linked to a higher frequency of ICU admission and re-intubation [22, 23], dementia, which is often associated with a higher risk of post-operative infectious complications, renal impairment and stroke [24] and atrial fibrillation (AF), which includes a new-onset of AF or worsened chronic AF [14].

### Tools for objectively and systematically defining postoperative ICU admission indications

**Question 2. What are the appropriate tools for objectively and systematically defining the indication for postoperative ICU admission?** Median score: 7 (IQR 7–9); agreement: 85.71%).

Statement 2.1: median score: 9 (IQR 5–9); agreement: 84.61%

Based on the current literature evidence, the most appropriate strategy seems to be to plan the ICU admission need for patients depending on the presence of multiple pre-existing conditions associated with several non-modifiable pre-existing factors (e.g., ASA score ≥ 3, Charlson Comorbidity Index ≥ 2, Rockwood Frailty Index ≥ 0.25, Revised Cardiac Risk Index (Lee Criteria) ≥ 2, BMI > 60, male gender, diabetes, sarcopenia) and/or in case of new or intra-operative events (e.g., Surgical APGAR score ≤ 7, intra-operative bleeding, hypotension requiring vasopressor support, respiratory issues) that require an intensive monitoring/treatment in the immediate post-surgery period.

Statement 2.2: median score: 9 (IQR 5–9); agreement: 92.3%

When predicting the need for ICU admission, the main factors to evaluate are the type of surgery and the patient’s comorbidities. Specific hospital organizations could contribute to this decision (e.g., availability of high dependency unit as step down ICU) (see flowchart in Supplementary information 1: Appendix 5).

Rationale

The increasing average age of the surgical population is a crucial factor to consider in the allocation of intensive care resources, since there are now always more elderly patients undergoing highly complex surgery [15, 25–27]. The greater the age, particularly after 80 years, the greater the need for post-operative intensive care assistance [26–28].

However, advanced age alone is not a sufficient criterion to define the indication for intensive care admission, which is necessarily influenced by the patient’s comorbidities, the type of surgical procedure, intra-operative blood loss, and possible new onset of peri-procedural complications [25]. In the geriatric setting, the predictive value of several frailty indexes on post-operative recovery after major surgery has been investigated.

For patients over 65, the presence of multiple comorbidities is a very important factor, evaluated with ASA score [15, 29–31], which shows a good correlation with ICU admission for patients undergoing, for example, gastrectomy, for scores ≥ 3 [29]. In non-cardiac surgeries, an increased percentage of ICU admission has also been reported in patients with Charlson Comorbidity Index ≥ 2 [15]. In major abdominal surgery, there was a demonstrated correlation between post-operative ICU admission and modified Rockwood Frailty Index [32] and frailty geriatric evaluation [33]. Furthermore, low intra-operative Bispectral Index (BIS) levels (< 35), reflecting low cerebral activity and often associated with the onset of post-operative delirium (POD), can cause unexpected ICU admission [34]. There is a close relationship between anaesthesia depth and hemodynamic. Hypotension can reflect low BIS values caused by low blood cerebral perfusion, but low BIS often indicates an anaesthetic overdose that may result in low blood pressure. Therefore, low BIS values may indicate organ hypoperfusion that may imply a worse outcome [35]. Nonetheless, in case of hemodynamic stability, the real need of ICU admission in POD patients is arguable, as it might hinder the application of non-pharmacological treatment strategies, “reorientation”, sleep–wake cycle regularization, early use of hearing or visual aids, as well as early contact with caregivers [35].

Among comorbidities, severe obesity [27, 30, 36], particularly with a BMI > 60 [37], and the OSAS presence [38], correlate significantly with ICU admission. Pre-operative malnutrition may also adversely affect the post-operative period. Literature shows an evident correlation between ICU transfer and the prognostic nutritional index [39], as well as pre-operative sarcopenia, which is associated with complications of grade ≥ 3, according to Clavien-Dindo Classification [40].

Both diabetes [19] and elevated pre-operative levels of suPAR (soluble urokinase plasminogen activator receptor), a marker of chronic inflammation, are significantly associated with the risk of ICU admission [41].

A systematic review demonstrated that CPET (cardiopulmonary exercise testing) is predictive of complications and unexpected ICU admission for non-cardiothoracic surgery [42], although other authors have found a significant predictive value only in hepatic surgery and liver transplantations [43].

In recent years, several tools to assess the risk of ICU admission have been investigated, including pre-operative [29, 44–47, 49] and/or intra-operative [48–50] risk factors. These include the Combined Assessment of Risk Encountered in Surgery (CARES), the Tanaka score, the Surgical Outcome Risk Tool (SORT), the Surgical Apgar Score, and several nomograms. All these tools are strongly correlated with ICU admission in major abdominal surgery.

Nonetheless, there is a lack in literature of comparative studies between the various scores and an accurate analysis of the potential impact of local organizations on ICU admission, such as the presence of PACU (post-anesthesia care unit), equipped for patients requiring high-intensity care or the presence of intermediate care units.

Finally, an advisable strategy remains to plan for ICU admission for ICU admission in case of more than one non-modifiable pre-existing factor (e.g., ASA score ≥ 3, Charlson Comorbidity index ≥ 2, Rockwood Frailty Index ≥ 0.25, BMI > 60, Revised Cardiac Risk Index (Lee Criteria) ≥ 2, male gender, diabetes, sarcopenia) and/or in case of new onset of intra-operatory occurrences (i.e., surgical APGAR score ≤ 7, intra-operative haemorrhage, hypotension requiring amine support, respiratory issues) that require intensive monitoring/treatment in the immediate post-operative period [29, 44–50]. Post-operative ICU admission, without univocal pre-operative predictive criteria, is mainly based on the patient’s comorbidities, the type of surgery, and the hospital’s organization [15, 51, 52].

Future studies should focus on the need to develop a tool that, objectively and systematically, suggests the potential need for post-operative ICU admission, considering the association of pre, intra and post-operative variables, in order to avoid inappropriate admissions and optimize available resources.

#### Tools to assess pre-operative frailty for determining postoperative ICU admission

**Question 3. What are the appropriate tools to assess the degree of frailty in pre-operative patients to determine post-operative ICU admission? **(median score: 8 (IQR 7–9); agreement: 12/14 (85.71%)).

Statement 3.1: median score: 9 (IQR 6–9); agreement: 100%

The evaluation of frailty conditions (as a multidimensional syndrome, prevalent among elderly patients, but not exclusively age-related, and significantly associated with adverse postoperative events) should be included in the pre-operative evaluation process.

Statement 3.2: median score: 9 (IQR 8–9); agreement: 100%

Although there is no high-quality evidence supporting a specific frailty assessment tool, the Clinical Frailty Scale (CFS) seems to be the most accurate and practical tool since it is fast and user friendly. These characteristics make it preferable for a routine approach in the pre-surgical evaluation process.

Statement 3.3: median score: 9 (IQR 6–9); agreement: 92.3%

The combination of frailty screening tests with risk assessment scores for postoperative complications (i.e., ACS NSQIP Surgical Risk) could provide a better estimation of the surgical risk and could guide decisions regarding post-operative monitoring in intensive or intermediate intensive environments. In elderly patients, in case of positivity to the frailty screening tests and before elective surgery that can be postponed, a geriatric multi-dimensional evaluation—which is also available in “short-form”—could be considered to identify possible peri-surgical optimization areas. Therefore, the use of a multidisciplinary agreement in each center would be useful to establish the tools to be used for a comprehensive pre-operative evaluation in order to improve the pre-operative clinical status, whenever possible.

Rationale

According to some future projections, within 2030, one-fifth of surgeries will be on patients over the age of 75 [53]. The increasing incidence of comorbidities in this cohort highlights the importance of continuously refining the pre-operative evaluation process in surgical candidates [54]. According to an international consensus, for an optimal management of frail patients, all patients over 70 and/or with significant weight loss (> 5%) in the last year due to chronic diseases should be screened for frailty [55]. Furthermore, it has been reported that frailty is consistently associated with at least a twofold increase in the risk of major complications, mortality, and hospital re-admission [56].

A recent meta-analysis highlighted that frail patients are not only at an increased risk of post-operative mechanical ventilation, prolonged ICU and hospital stays, but also have higher short-term (intra-operative, ICU, in-hospital, and 30 days) and long term (≥ 6 months) mortality [57].

Nonetheless, there has been an increasing interest in frailty study for candidates to surgeries in order to stratify their overall operative risk more precisely. In fact, for what concerns surgical patients, the progressive age-related decline and the gradual exhaustion of physiological reserve, identified by their degree of frailty, lead to a lower resilience and a reduced ability to adapt to surgical stress and, consequently, to major post-operative vulnerability [58–62]. Although there is now a huge agreement in literature regarding the definition of frailty and the importance of its identification and quantification in the surgical setting, there is no consensus, also due to poor quality studies on which score or specific tool could be preferred to identify and measure the frailty status of surgery candidates [63, 64].

An ideal frailty evaluation tool should meet criteria for predictive accuracy, feasibility and ease of use to avoid overburdening an already complex pre-operative screening, where time is a limiting factor. The most well-studied multidimensional evaluation tools in pre-operative surgical setting are the Frailty Phenotype (FP), the Clinical Frailty Scale (CFS), the Frailty Index (FI), and the Edmonton Frail Scale (EFS). In literature regarding major non-cardiac surgery, the predictive accuracy of several scales does not differ significantly. For example, a comparison between CFS and FP showed no significant differences in terms of sensitivity and specificity for risk stratification of mortality or new disability following elective non-cardiac surgery [65]. However, few studies made a direct comparison between different frailty scores and assessment scales, crucial for establishing their predictive accuracy, discrimination power, and calibration [66]. A recent study on elective non-cardiac surgery showed that CFS has a better discriminatory ability for mortality or the development of new disabilities compared to FP or FI, when included in a pre-operative risk stratification model that also comprises age, sex, ASA classification, surgery type [66]. Moreover, in terms of speed and feasibility, CFS seems to be the best tool [65].

The ability to predict outcomes in frail patients can be improved by merging frailty evaluation tools, with Surgical Apgar Score or ACS NSQIP Surgical Risk. By incorporating more variables, these combinations can provide a more reliable index of short and medium-long term operative risk [62, 64]. This approach is not without organizational and procedural challenges. Given the favourable evidence available, every hospital, especially referral centers where the most complex cases converge, should integrate frailty evaluation into their pre-hospitalization pathway [64].

Finally, the current literature does not clarify yet the true potential of tailored interventions, including multidimensional approaches, to mitigate or alleviate frailty, and its consequences, before surgery [67].

#### Tools to assess surgical impact and technique for determining the need for postoperative intensive or sub-intensive care admission

**Question 4. What tools allow to assess the surgical impact and/or surgical technique on the single patient’s physiology in order to establish whether there is an indication for post-operative intensive/sub-intensive care unit admission?** (median score: 9 (IQR 8–9); agreement: 13/14 (92.86%)).

Statement 4.1: Median score: 9 (IQR 8–9); Agreement: 100%

The intrinsic risk associated with surgical procedures is a predicting factor for post-operative outcomes. The several pre-operative scores elaborated to estimate the surgical risk include even variables linked to patient characteristics (e.g., Apgar Surgical Score, ACS NSQIP Surgical Risk). Therefore, the impact of the surgical risk cannot be separated from patient-specific characteristics.

Statement 4.2: median score: 8 (IQR 1–9); agreement: 76.92%

Most surgical risk scores currently available do not include the surgical approach nor a pre-operative optimization. Minimally invasive techniques (laparoscopic, robotic-assisted) and multimodal prehabilitation (according to enhanced recovery after surgery (ERAS) programs) should be encouraged, when possible, to reduce the surgical impact on frail patients and/or patients with organ dysfunctions.

Rationale

The intrinsic risk associated with surgery is one of the post-operative course predictors (along with the patient’s pre-operative risk factors and post-operative complications). Surgical risk depends on the extent and location of the surgery, blood loss, and fluid shifts to the third space, hemodynamic effects (hemodynamic stress), and duration [68]. The Surgical Apgar Score allows to estimate the patient’s risk only at the end of the surgery, based on intra-operative variables [48].

Some pre-operative scores are based on risk factors that may have a negative impact on the post-operative course. Among these, one of the most used is the ACS NSQIP Surgical Risk, which is based on 21 pre-operative variables, most of which are related to the patient’s characteristics. Nonetheless, the impact of the surgical risk cannot be separated from the specific patient’s characteristics, thus there are scores that merge the two elements [69].

Minimally invasive surgical techniques result in lower surgical trauma, and thus reduce the risk associated with surgery. The goal of these techniques is to perform specific operations while minimizing surgical trauma. In abdominal surgery, for example, there is a reduction in incision numbers and fewer organ manipulations, leading to a consequent reduction in inflammatory response and immune dysfunction. There is also a lower impact on the pulmonary function, with a lower incidence of hypoxemia, compared to open surgery. Post-operative paralytic ileus is also reduced.

Indeed, many studies report less pain, shorter post-operative course, less ICU admissions and reduced perioperative rate of mortality and morbidity in patients undergoing minimally invasive surgical procedures [70–73].

Minimally invasive surgical techniques are a crucial component of ERAS (enhanced recovery after surgery) and FAST TRACK protocols. Thus, these procedures are based on the concept of multimodal peri-operative interventions, which reduce post-operative organ dysfunction and consequent morbidity [74].

#### Criteria and assistance profiles for postoperative monitoring and support: defining intermediate assistance levels between the surgery ward and ICU

**Question 5.** **Can some criteria or assistance profiles be identified for postoperative monitoring and support in order to define intermediate assistance levels between the surgery ward and ICU?** (Median score. 8 (IQR 7.25–9); Agreement: 85.71%

Statement 5.1: median score: 9 (IQR 5–9); agreement: 76.92%

For certain high-complexity surgical disciplines, it may be beneficial to establish units with higher intensity of care than standard surgical wards, such as high-dependency units where continuous monitoring of vital functions is provided. The fundamental strategies to optimize post-operative courses are patient selection and staff training.

Statement 5.2: median score: 9 (IQR 5–9); agreement: 84.61%

To define the level of post-operative assistance for each patient, it is necessary to consider the following:

1. Age, comorbidities, and risk factors for metabolic and organ complications
2. Type, invasiveness, and duration of surgical procedure
3. Unexpected intra-operative events
4. The clinical and metabolic patient’s conditions upon awakening and the onset of immediate postoperative complications, including POD
5. Available facilities and resources (including human ones)

Rationale

Post-operative recovery is a crucial phase for every surgical patient, as it can influence the outcome of the surgery [75]. Improvements in surgical and anesthesiological knowledge, technologies, procedures, and drugs, combined with experience, have led to an increase in the number and complexity of surgery. This expansion has also broadened surgical options to encompass larger patient populations, including elderly individuals with comorbidities [76]. Consequently, the monitoring and protection of the patient’s vital functions following surgery in order to provide reasonable safety and outcome standards have become increasingly important [76, 77].

These interventions have collectively led to a progressive paradigm shift in the mindset of the post-operative approach to the surgical patient. Thus, there has been a transition from a “reactive” management of post-operative complications (i.e., treating complications as they occur) to a pro-active model that emphasizes pre-planned post-operative care and prevention as a cornerstone [77].

From this perspective, a key role is played by the development of personalized post-operative care plans, tailored differently in terms of care aspects, logistics and organization based on the patient’s characteristics and the kind of surgery [78].

Basically, post-operative patient management could require different types of assistance and monitoring (respiratory, cardiovascular, neurological, metabolic) [77].

In order to provide useful practical guidelines for the decision-making process, aimed at selecting the most appropriate post-operative care setting based on the patient’s needs, the Society of Critical Care Medicine (SCCM) defined intermediate care units (IMCU), over 30 years ago. The purpose of these units is to monitor and take care of patients with moderate or potentially severe instability, requiring a lower level of care than that given by qualified and diligent personnel of ICU wards, though still requiring more qualified and attentive medical and nursing care than that given in a standard ward [79].

Conditions that may require admission to IMCU after major abdominal surgery include the following:

- Patients who are hemodynamically stable but require volume and transfusions resuscitations.
- Patients who are hemodynamically stable but require gradual weaning from mechanical ventilation.
- Patients in spontaneous breathing, hemodynamically stable but with altered gas exchange, requiring frequent observation and/or non-invasive ventilation or high flow oxygen therapy.
- Patients requiring continuous monitoring, including non-invasive vital signs monitoring, and/or management of drains, requiring diligent nursing assistance in the first 24 h after surgery for potential surgical and/or medical complications, without the need of intensive treatments.
- Patients with chronic conditions, requiring immediate post-operative treatments too complicated for a standard surgical ward (continuous renal replacement therapy (CRRT), continuous positive airway pressure (CPAP), cardiovascular support with cardio/vasoactive drugs).

Most studies focus on monitoring immediate post-operative respiratory functions since bradypnea and desaturation are strongly associated with the development of respiratory complications, especially in case of pre-existing risk factors such as age, respiratory diseases, smoking, and opioid administration [80]. More specifically, a baseline or arrival SpO2 < 96% in the PACU, associated with risk factors, may require oxygen therapy, BiPAP, or invasive ventilation [81].

Other authors have assessed the correlation between respiratory complications and integrated pulmonary index, which include EtCO2, heart rate, as well as SpO2 and respiratory rate, in patients at risk due to advanced age (> 75) and/or elevated BMI (> 28) [82]. Episodes of desaturation are often associated with the duration of surgery [83]. Nonetheless, a systematic review by Cochrane, involving over 22,000 patients undergoing non-abdominal surgery, confirmed that routine continuous pulse oximetry monitoring can detect episodes of hypoxemia and related events, without influencing outcomes such as ICU transfers and mortality [84].

Some studies focus on the importance of monitoring delirium’s signs [85] and prevention strategies [86] as delirium is associated with prolonged hospital stays, including in the ICU, and worse prognosis [87].

Likewise, great importance should be given continuous monitoring of blood pressure and cardiac rate since hypotension and tachycardia can be associated with development of severe adverse events [87].

Currently, there is no evidence directly comparing different post-operative care settings (i.e., ICU vs IMCU vs PACU, vs standard ward) in terms of outcomes. Moreover, international variations in organizational models and even the definitions of these care settings complicate the design of definitive and consistent conclusions on this matter. Thus, it is important that each hospital with surgical activities—especially referral HUB hospital, where higher complexity cases converge—to initiate a clinical governance in order to identify appropriate post-operative pathways, tailored to the patients’ typologies and the surgeries typically managed in the facility. This approach ensures the delivery of best practice and performance, and the merging of adequate clinical management and meticulous organizational management [88].

In referral hospitals, identifying dedicated multidisciplinary teams for the peri-operative care pathway of high-risk patients can be beneficial to provide daily monitoring until discharge and possibly for comprehensive follow-up [89].

#### Parameters to reassess at end of surgery and before PACU discharge to confirm or modify ICU admission indications

**Question 6. Which parameters could be reassessed at the end of surgery and before PACU discharge to confirm, indicate, or modify indication for ICU admission?** (median score: 9 (IQR 8–9); agreement: 92.86%

Statement 6.1: median score: 9 (IQR 5–9); agreement: 84.61%

The clinical feature of the patient in the immediate post-operative period is closely related to pathophysiological interactions between pre-operative clinical conditions, surgery impact and anesthesia on organs and systems, mechanical ventilation effects, fluids, blood and hemoderivates administered.

Statement 6.2: median score: 9 (IQR 3–9); agreement: 84.61%

Mild-high-risk patient observation in PACU is crucial for post-operative management as it allows for the evaluation and monitoring of vital parameters and immediate treatment. It could represent a transitory phase for the patient’s treatment, before establishing ICU transfer, depending on neurological status, cardiorespiratory and renal functions, metabolic compensation, and possible immediate post-operative complications. Score utilization at discharge could support a comprehensive evaluation of the patient.

Statement 6.3: median score: 9 (IQR 1–9); agreement: 92.3%

Neurological state and/or cardiovascular/respiratory function alterations are determining factors for re-evaluating the patient's destination. The concomitant use of scores and checklists, as well as clinical judgment, can make one decide for the most appropriate intensity level of care required by the patient.

Statement 6.4: median score: 9 (IQR 3–9); agreement: 84.61%

Organ and/or metabolic dysfunction of moderate to high grade, even if the patient is stable, may require continuous post-operative monitoring, feasible only in high assistance level settings. For this reason, hospital Governance should encourage the development of differentiated care pathways for surgical patients, based on single missions and local resources.

Rationale

PACU is a critical component in the management of the immediate post-operative period. It is a specially equipped area for patient assistance, where continuous monitoring of vital signs, pain management and treatment are conducted Literature supports the use of post-anaesthesia scores and checklists to evaluate the appropriate destination for the patient in the immediate post-operative period [90].

The discharge scores from the PACU are inspired by the historical White score and the modified Aldrete score. They are generally related to the recovery of consciousness, the stability of respiratory and haemodynamic functions, and the initial recovery from any loco-regional anaesthesia [91].

In literature, studies have been published on other validated, and more comprehensive scores, which consider also other parameters, such as pain, post-operative nausea and vomiting, temperature, and discomfort. Among these, the ESS (Efficacy Safety Score) although showing lower specificity compared to MEWS (Modified Early Warning Score) in identifying patients at risk of criticality, allows for a relatively straightforward assessment of the patient’s post-operative condition. It includes aspects related to the quality and safety of care providing an alarm cut-off (i.e., ESS S ≥ 10 or 15) for further medical evaluation [92].

The Simplified Transfer Checklist (SAMPE) evaluates the main transfer criteria [91]:

1. Stable vital signs
2. Neurological integrity or recovery of pre-operative sensorium
3. Spontaneous breathing
4. SpO2 > 90%
5. Pain control
6. Absence of nausea and vomiting
7. Absence of bleeding, and
8. Absence of motor block

The use of opioids, hypnotics, and neuromuscular blockers in general anaesthesia, as well as mechanical ventilation, lead to a risk for postoperative complications, particularly respiratory ones. During the early stages of anaesthesia recovery, potentially high-threatening respiratory complications (i.e., hypoxemia, hypoventilation, airway obstruction, and residual neuromuscular block) may occur, leading to respiratory failure.

Recent literature emphasizes this aspect, and new several scores have been developed to identify patients at risk.

The Air-Test score is a simple test, based on measuring SpO2 in ambient air (negative cut-off if SpO2 ≥ 96%) before surgery, and three hours post-PACU admission. It stratifies patients in four risk grades for respiratory complications: low risk (pre and post-tests negative) with ≤ 15% probability of post-operative pulmonary complications; low-intermediate risk with 15–30% probability; intermediate risk with high probability 30–50%; high risk with probability ≥ 50–75% for respiratory complications [93].

Schumann et al., using an electronic monitoring system in PACU to measure tidal volume, minute ventilation, and respiratory rate, identified patients at risk of developing opioid-induced respiratory depression in the first 12–24 h post-surgery [94].

There is evidence in literature showing a correlation between opioid-induced respiratory depression and subsequent adverse respiratory events in general care units.

Identifying episodes of respiratory depression in PACU allows for better stratification of the risk of respiratory adverse events, especially in patients with other risk factors, such as OSAS or cardiovascular or respiratory diseases, elderly patients, patients with cognitive impairment, and under chronic treatment with opioids or gabapentinoids [95].

Kuroe et al. evaluated the predictive value for post-operative respiratory issues in elderly (≥ 75 years) and/or obese patients (BMI ≥ 28), using the IPI (Integrated Pulmonary Index), which is calculated based on four parameters: etCO2, respiratory rate, SpO2, heart rate. The IPI classifies patients with a score of 10 points: ≥ 8 indicating a normal range, ≤ 4 indicating the need for medical evaluation/intervention [82].

The retrospective analysis of Rostin’s study on several thousands of patients highlighted a correlation between desaturation events (SpO2 ≤ 90%) within the first 10 min post-extubation and the need for post-operative care in a higher-intensity environment. Furthermore, it revealed modifiable risk factors for desaturation, such as intra-operative administration of long-acting opioids, high doses of neostigmine, high intra-operative FiO2, and low pre-extubation FiO2 [96].

Prolonged delirium throughout the observation phase in PACU, is associated with post-operative complications; thus, it could be a decisive factor in determining the patient’s destination [97].

## Conclusion

The evaluation of patient characteristics, comorbidities, and surgical factors are essential for an adequate planning of ICU admission for immediate postoperative care after elective major abdominal surgery.

The presence and severity of comorbidities, assessed through various severity scores, play a crucial role in predicting postoperative complications and guiding ICU admission decisions. Tools such as the ASA score, Charlson Comorbidity Index, and Rockwood Frailty Index, along with surgical risk scores and intraoperative events, help define the need for intensive care.

Frailty assessment, particularly using the Clinical Frailty Scale, is vital in preoperative evaluation to anticipate postoperative care needs. This tool, combined with surgical risk scores and strategies such as minimally invasive techniques and ERAS programs, can mitigate the impact of surgery on vulnerable patients.

Finally, during the postoperative phase, continuous monitoring, and reassessment in the PACU are crucial to determine whether ICU admission is required. Vital signs, neurological and cardiovascular status, and the overall clinical picture evaluation ensure the appropriate allocation of care resources. Establishing high-dependency units and tailored care pathways based on individual patient needs and available resources will enhance patient outcomes and optimize postoperative care.

## Supplementary Information

Supplementary Material 1: Appendix 1. Results of the 1st round of voting: clinical queries. Appendix 2. Results of the 2nd round of voting: clinical queries. Appendix 3. Results of the 1st round of voting: statements and rationales. Appendix 4. PRISMA flow. Appendix 5. Proposal of decisional algorithm.

## Data Availability

No datasets were generated or analysed during the current study.
