# Supplementary material for: Planning intensive care unit admission after elective major abdominal surgery: good clinical practice document by SIAARTI-SIC-ANIARTI
Source: J Anesth Analg Crit Care. 2025 Apr 14;5:20. doi: 10.1186/s44158-025-00239-w (PMC11995668; doi:10.1186/s44158-025-00239-w)
Supplement: Supplementary file 1 — Supplementary Material 1: Appendix 1. Results of the 1st round of voting: clinical queries. Appendix 2. Results of the 2nd round of voting: clinical queries. Appendix 3. Results of the 1st round of voting: statements and rationales. Appendix 4. PRISMA flow. Appendix 5. Proposal of decisional algorithm. [file 44158_2025_239_MOESM1_ESM.docx]

**APPENDIX 1 -** **RESULTS OF THE 1^ST^ ROUND OF VOTING:** **CLINICAL QUERIES**

|  | | **Not Relevant** | | **Uncertain** | | **Relevant** | |
| --- | --- | --- | --- | --- | --- | --- | --- |
| 1. **What are the patient’s characteristics/comorbidities to objectively and systematically define the appropriateness of ICU admission?** | | | | | | | |
| Median score. 9 (IQR 7,25-9);  **Agreement: 13/14 (92,86%)** | | 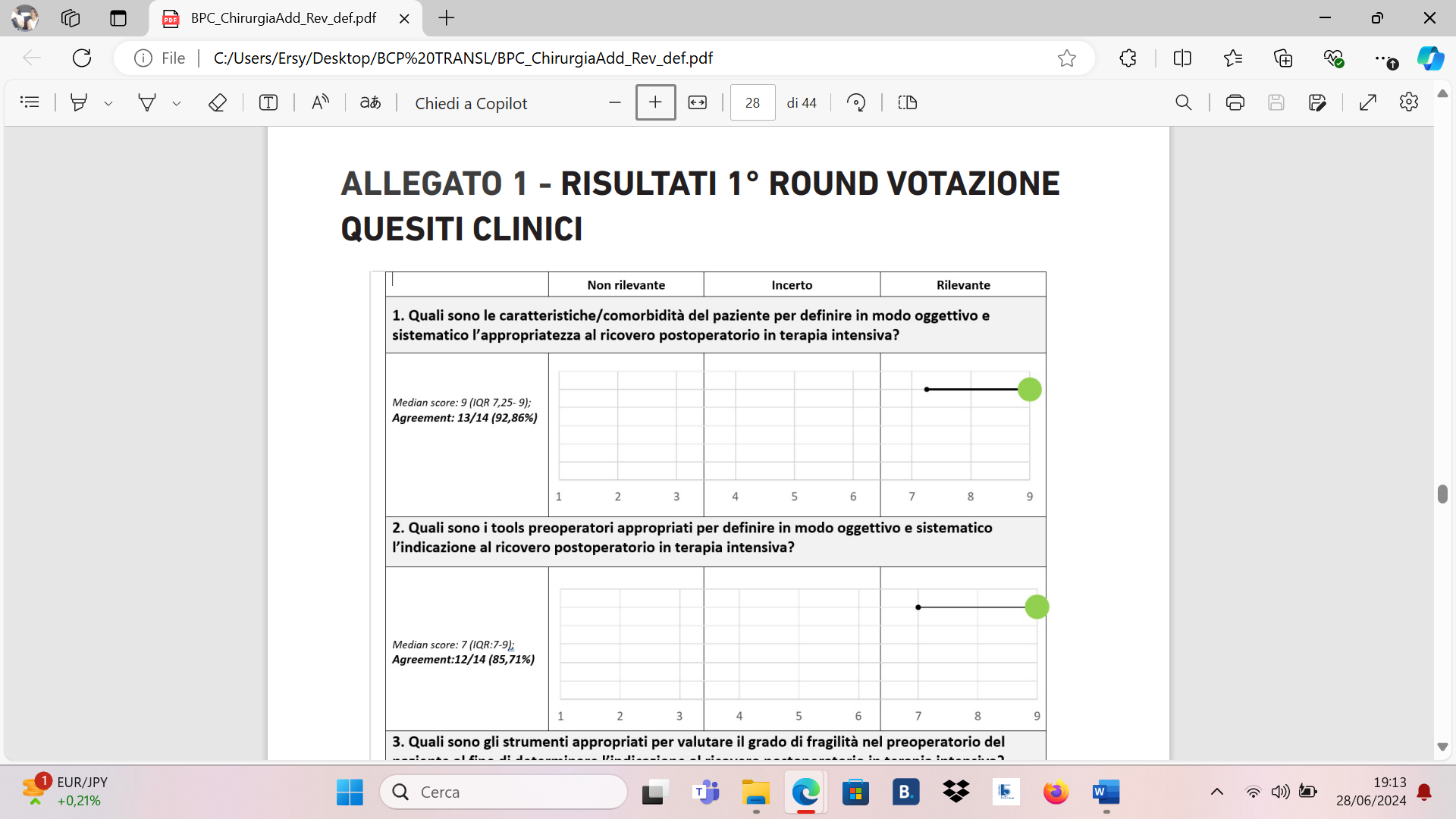 | | |  | |  |
| 1. **What are the appropriate tools for objectively and systematically defining the indication for postoperative ICU admission?** | | | | | | | |
| Median score: 7 (IQR 7-9);  **Agreement: 12/14 (85,71%)** | 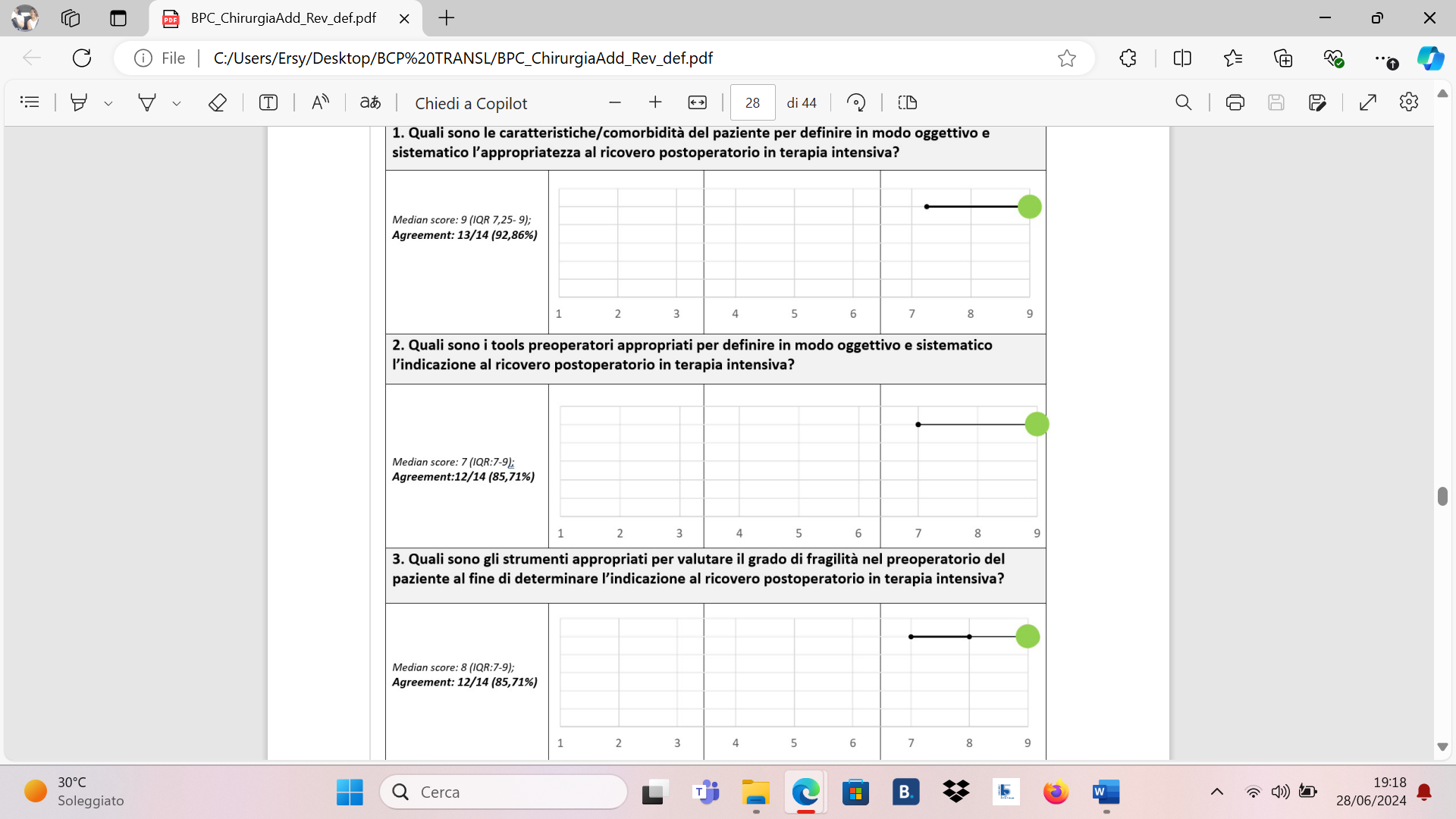 | | |  | |  | |
| 1. **What are the appropriate tools to assess the degree of frailty in pre-operative patients in order to determine post-operative ICU admission?** | | | | | | | |
| Median score: 8 (IQR 7-9);  **Agreement: 12/14 (85,71%)** | | 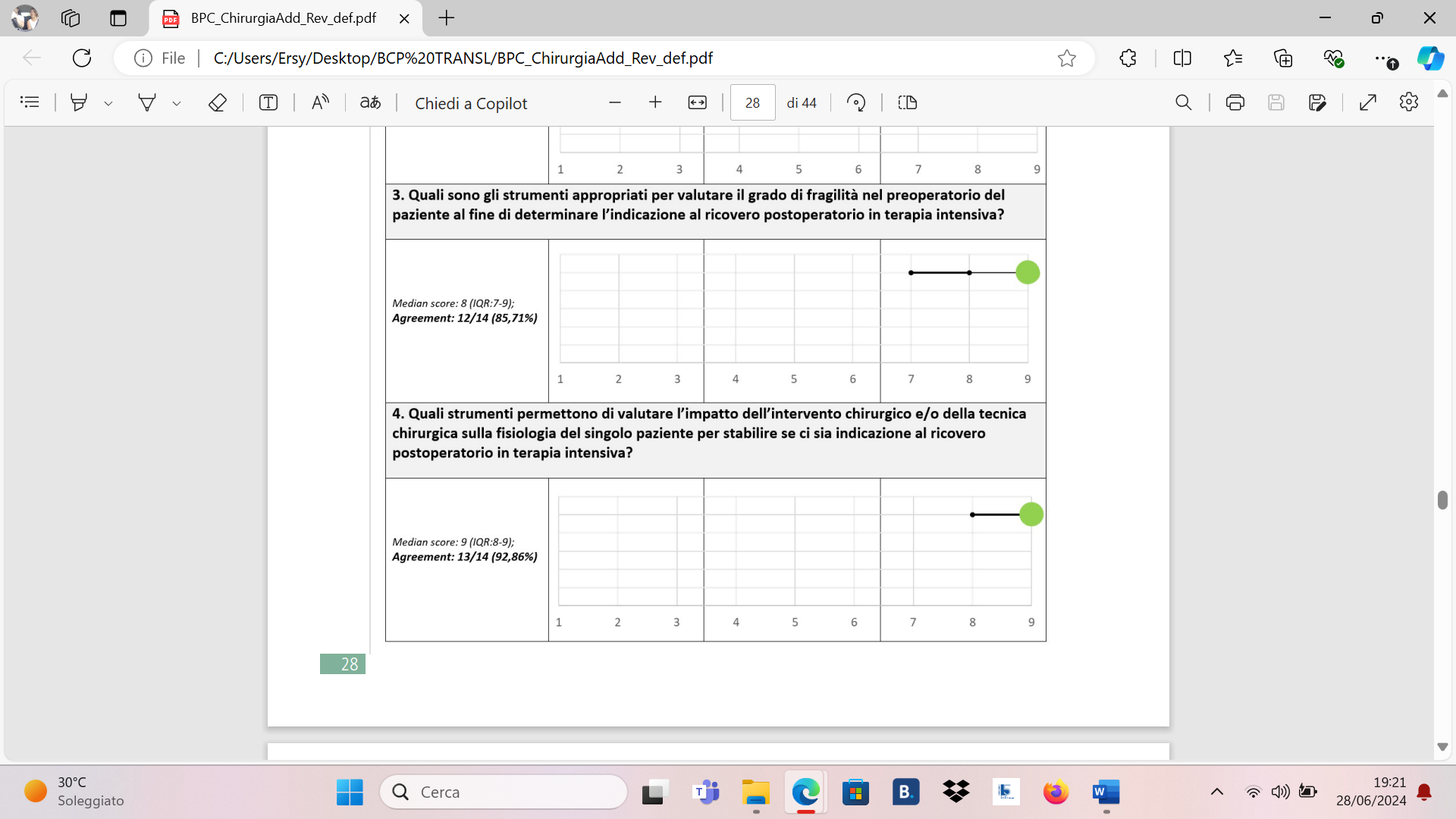 | |  | |  | |
| 1. **What tools allow us to assess the surgery impact and/or surgical technique on the single patient’s physiology in order to establish whether there is an indication for post-operative intensive/sub-intensive care unit admission?** | | | | | | | |
| Median score: 9 (IQR 8-9);  **Agreement: 13/14 (92,86%)** | | 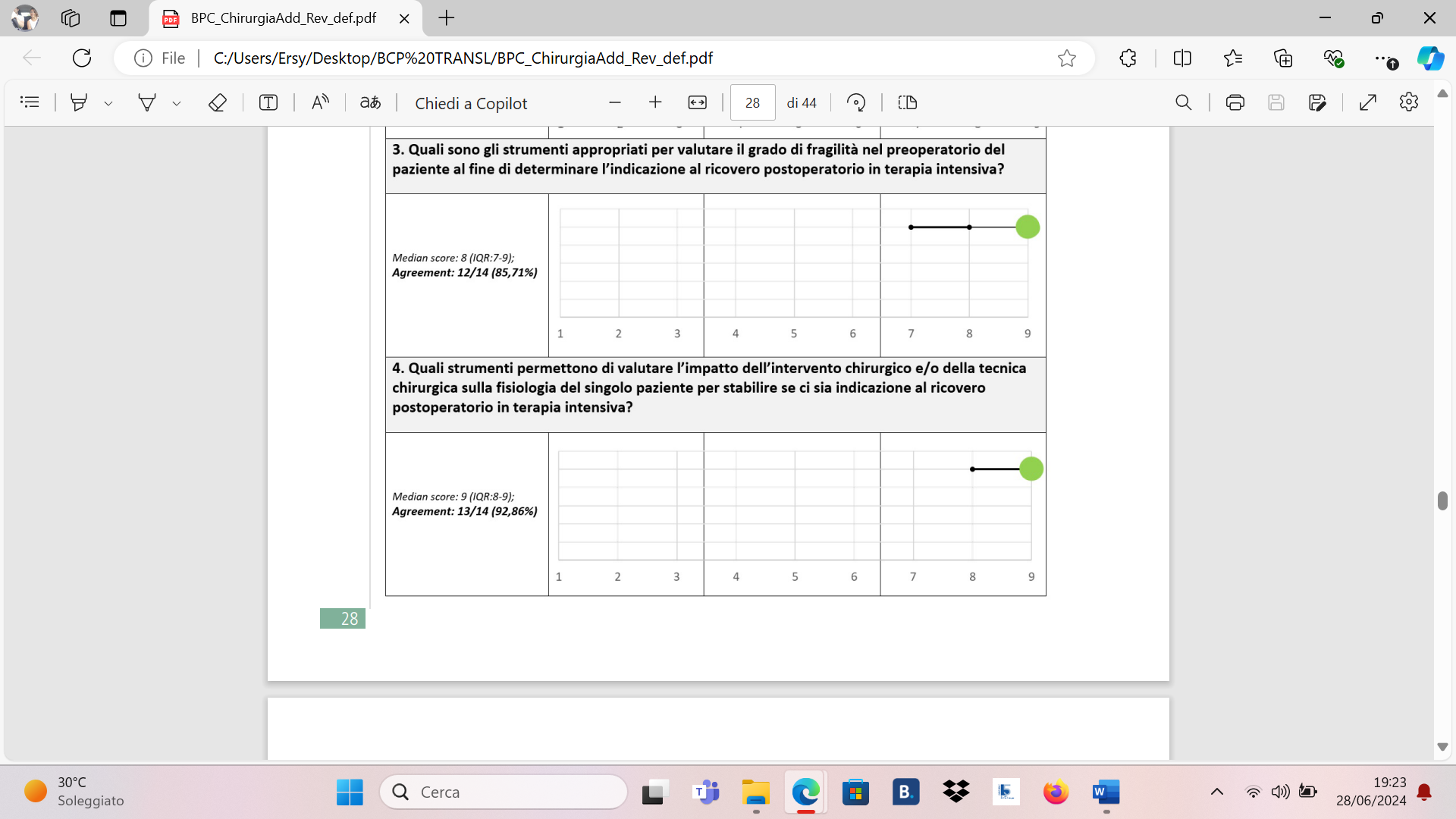 | |  | |  | |
| 1. **What tools can be used to assess the impact of the anaesthetic technique and its management on the patient's physiology to determine if there is an indication for postoperative admission to the intensive care unit?** | | | | | | | |
| Median score: 7,5 (IQR 5-8,75);  **Agreement: 8/14 (57,14%)** | | 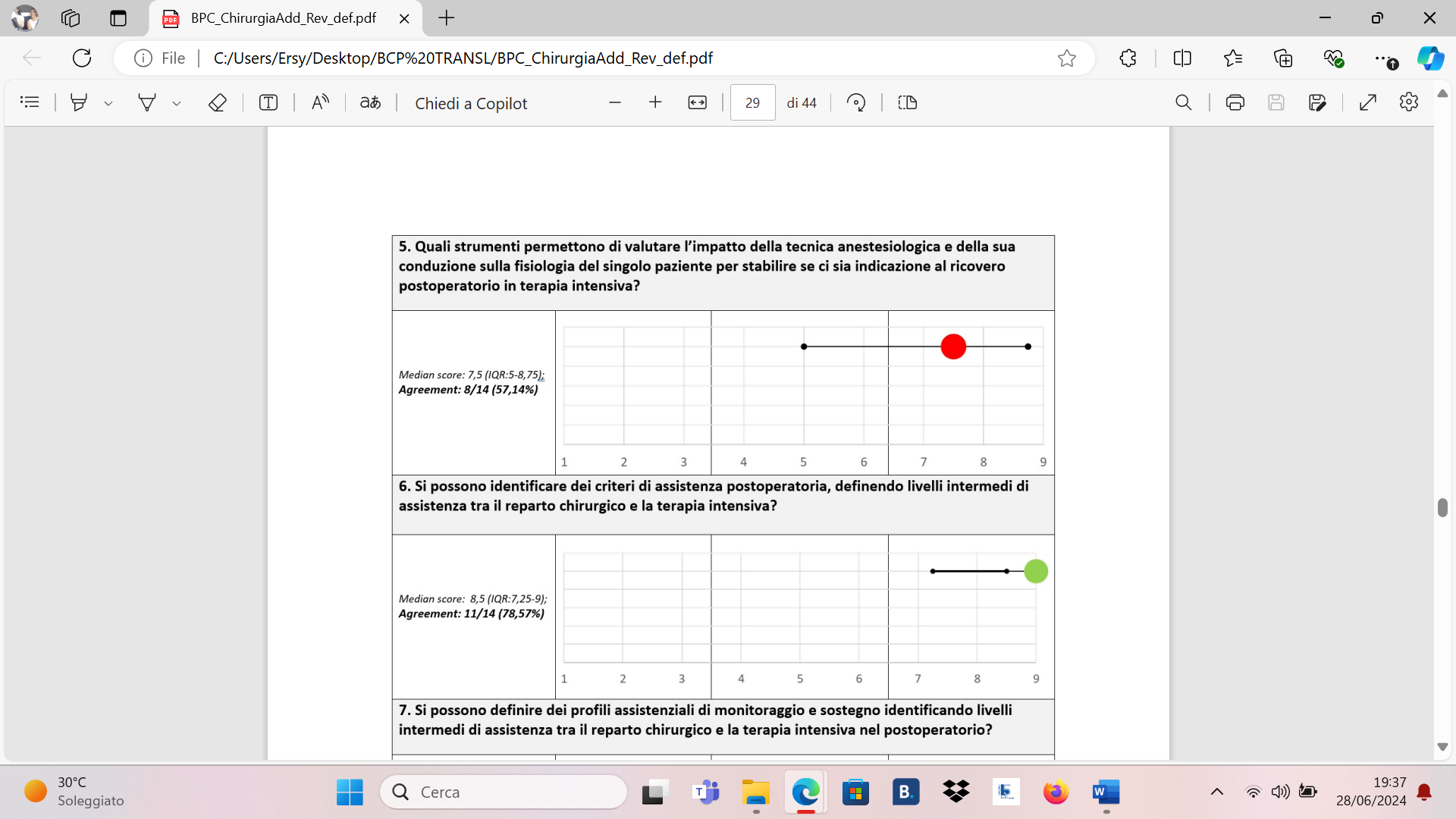 | |  | |  | |
| 1. **Is it possible to identify post-operative care criteria by defining intermediate levels of care between the surgical ward and the intensive care unit?** | | | | | | | |
| Median score. 8,5 (IQR 7,25-9);  **Agreement: 11/14 (78,57%)** | | **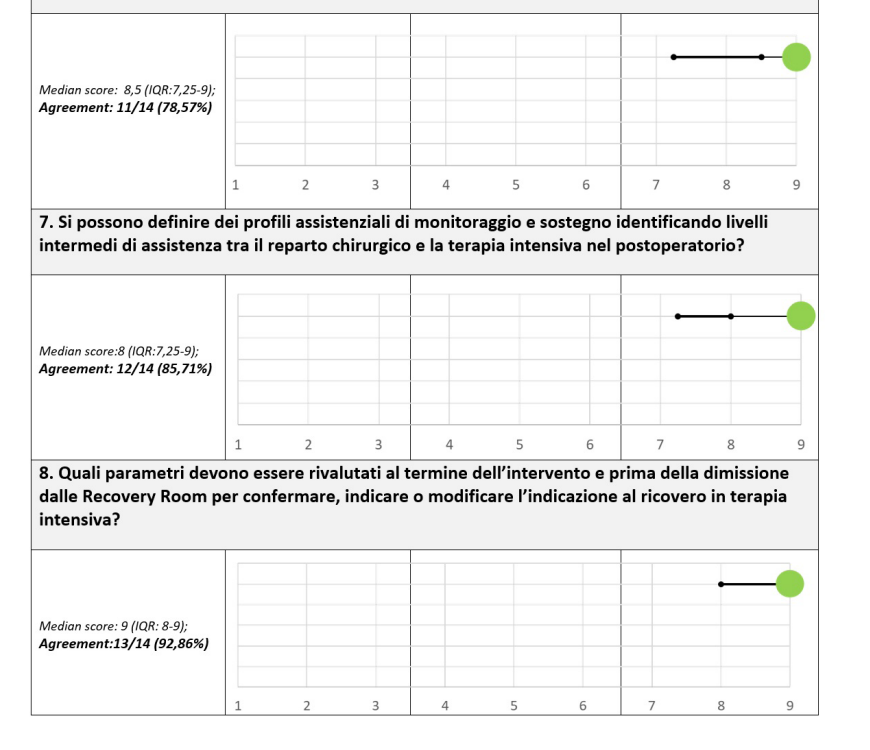** | |  | |  | |
| 1. **Can some criteria or assistance profiles be identified for postoperative monitoring and support in order to define intermediate assistance levels among the surgery ward and ICU?** | | | | | | | |
| Median score. 8 (IQR 7,25-9);  **Agreement: 12/14 (85,71%)** | | **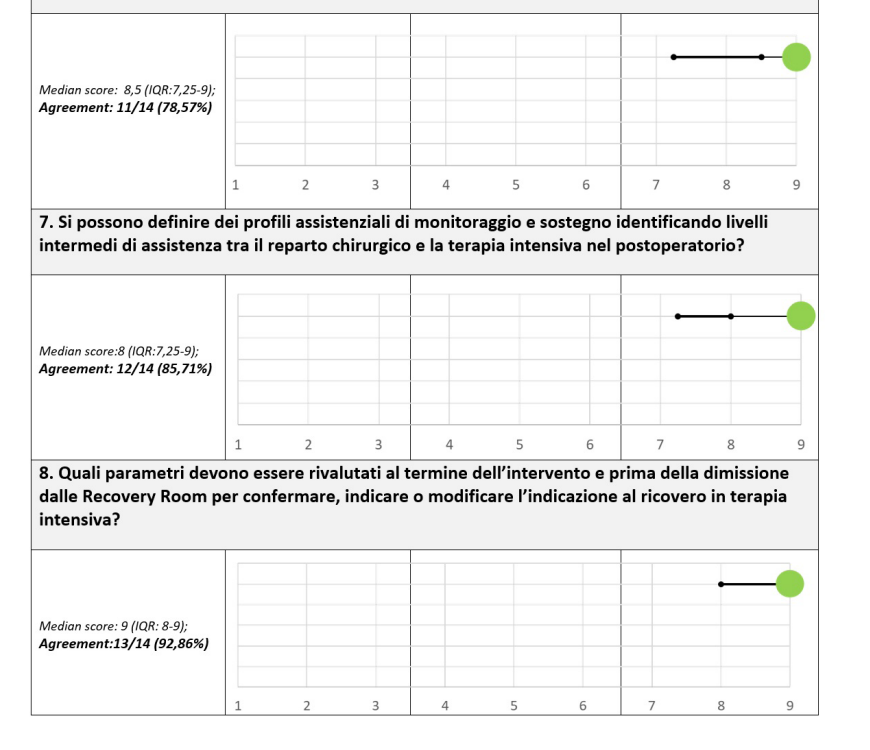** |  | | |  | |
| 1. **Which parameters could be reassessed at the end of surgery and before PACU discharge to confirm, indicate, or modify indication for ICU admission?** | | | | | | | |
| Median score: 9 (IQR 8-9);  **Agreement: 13/14 (92,86%)** | | **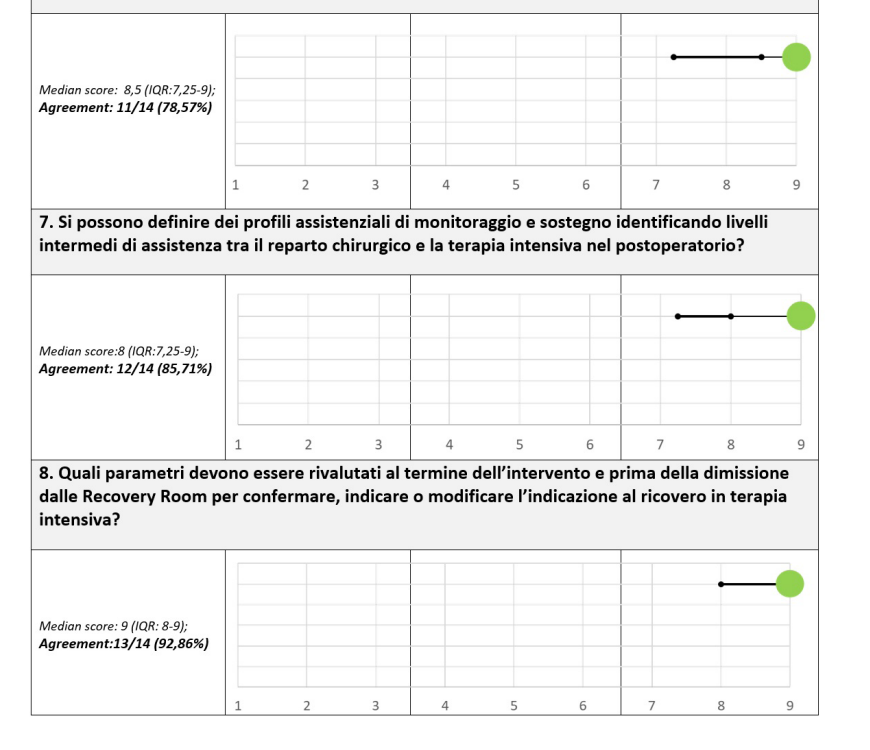** | |  | |  | |

**APPENDIX 2 - RESULTS OF THE 2^nd^ ROUND OF VOTING:** **CLINICAL QUERIES**

|  | **Not Relevant** | **Uncertain** | **Relevant** |
| --- | --- | --- | --- |
| **Are there anaesthetic treatment protocols that can impact the physiology of the individual patient and modify the indication for post-operative admission to the intensive care unit?** | | | |
| Median score: 8 (IQR 6,25-8);  **Agreement: 10/14 (%)** | 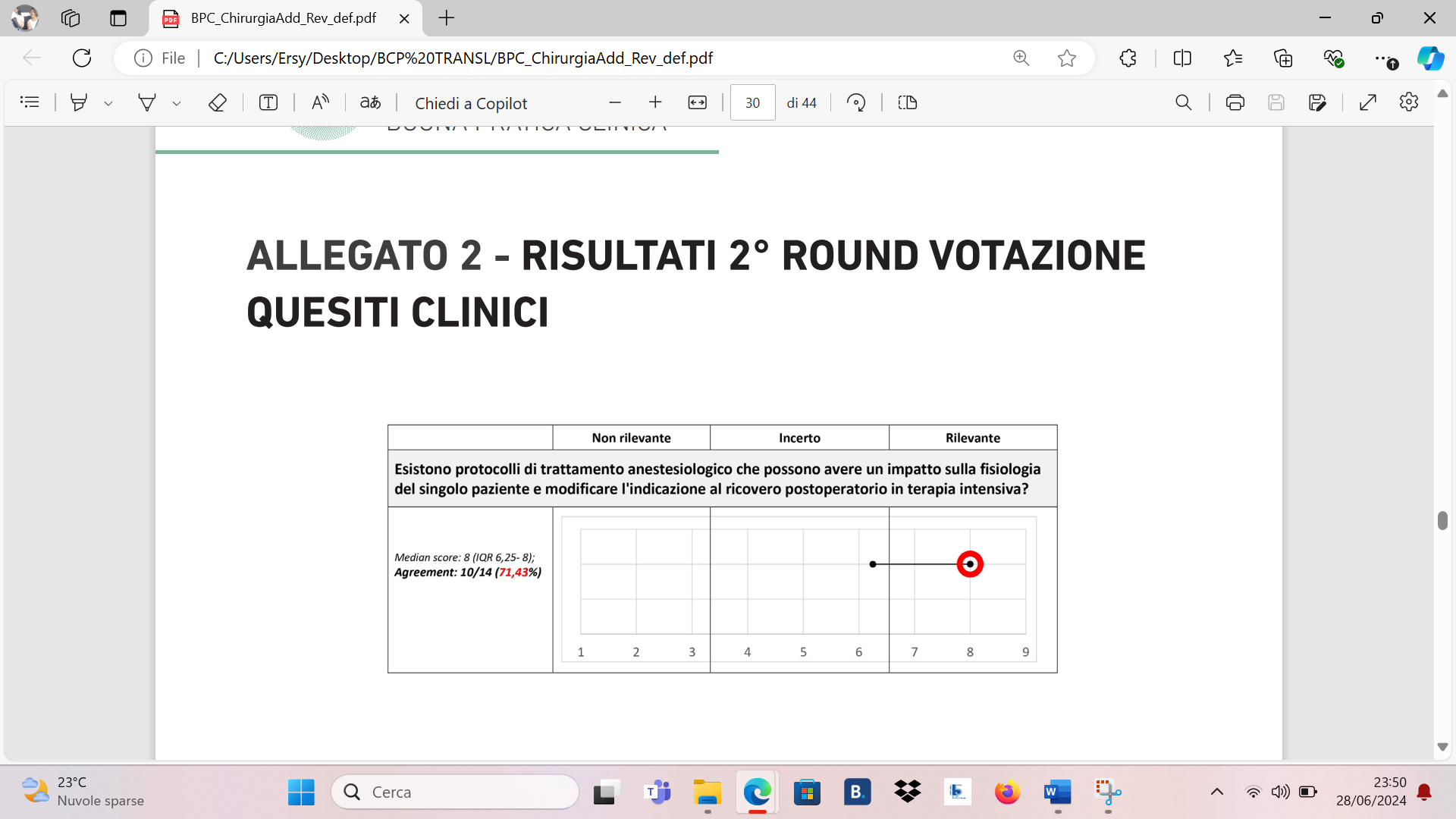 |  |  |

**APPENDIX 3 - RESULTS OF THE 1st ROUND OF VOTING:** **STATEMENTS AND RATIONALES**

|  | **Not Relevant** | **Uncertain** | **Relevant** |
| --- | --- | --- | --- |
| **1.1 The number of comorbidities, i.e., the presence of multiple comorbidities, combined with patient characteristics, increases the risk of postoperative complications. However, this risk also depends on factors occurring intraoperatively and in the immediate postoperative period.** | | | |
| Median score: 9 (IQR 6-9);  **Agreement: 11/13 (84,61%)** | 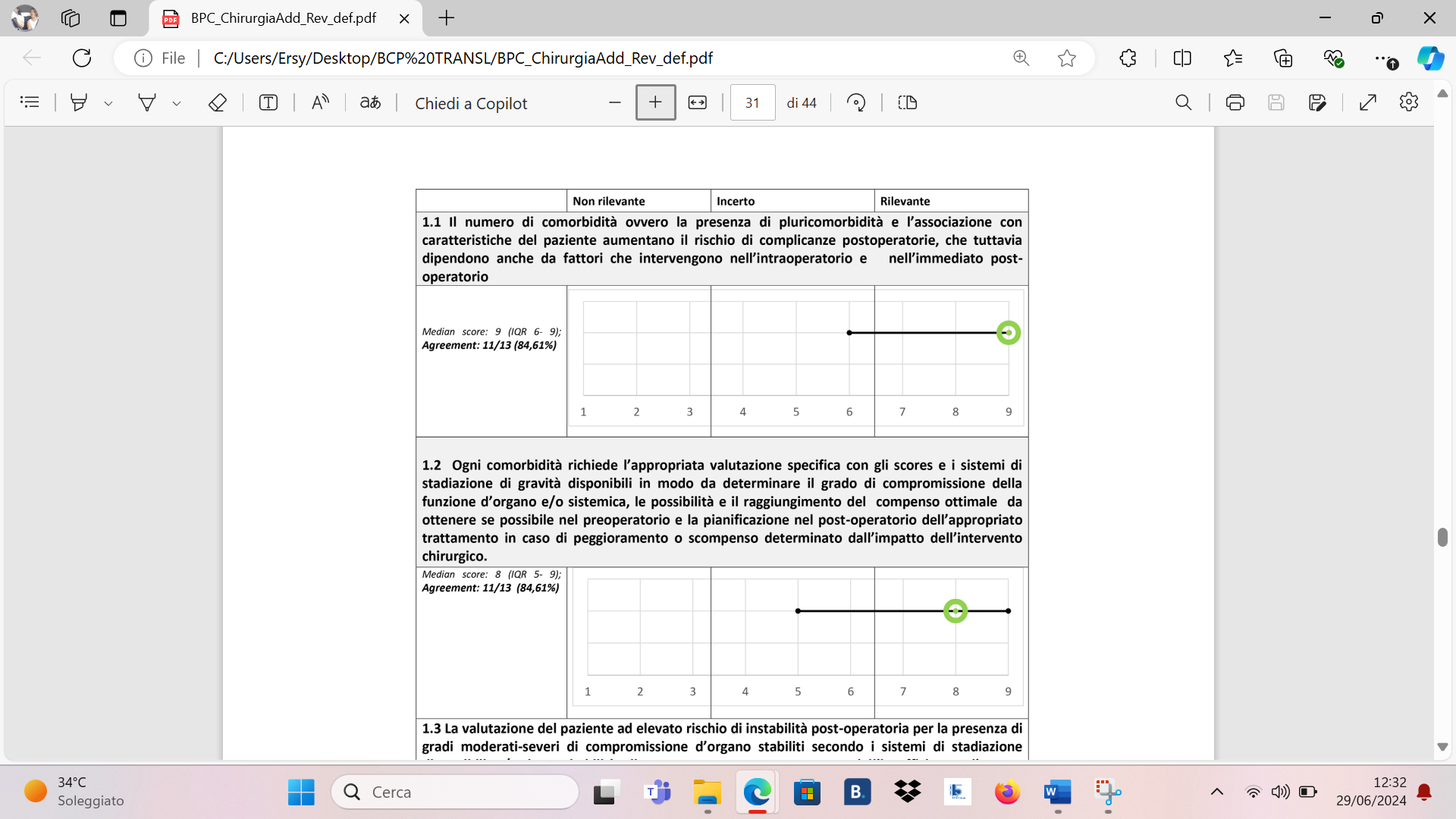 |  |  |
| **1.2 Each comorbidity requires appropriate specific evaluation with available severity staging scores and systems to determine the degree of organ and/or systemic dysfunction, the possibilities, and the achievement of optimal compensation, if possible, in the preoperative period, as well as the plan of appropriate postoperative treatment in case of deterioration or decompensation due to the impact of the surgical intervention.** | | | |
| Median score: 8 (IQR 5-9);  **Agreement: 11/13 (84,61%)** | 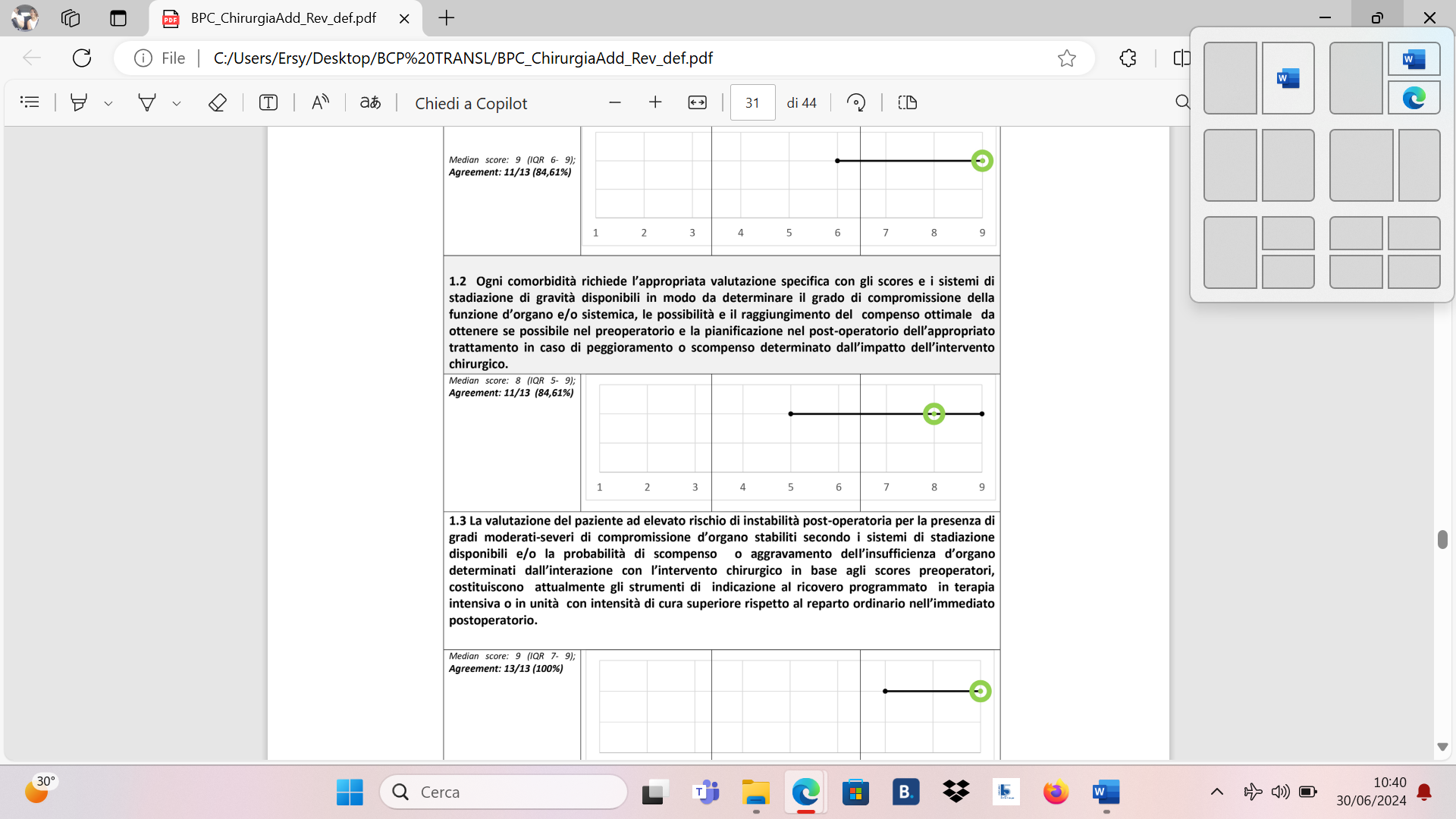 |  |  |
| **1.3 The assessment of high-risk patients for postoperative instability, due to the presence of moderate to severe organ impairment as determined by available staging systems and/or the likelihood of organ failure or worsening determined by interaction with the surgical procedure based on preoperative scores, currently constitutes the criteria for planned admission to intensive care units or units with a higher level of care compared to the standard ward in the immediate postoperative period.** | | | |
| Median score: 9 (IQR 7-9);  **Agreement: 13/13 (100%)** | 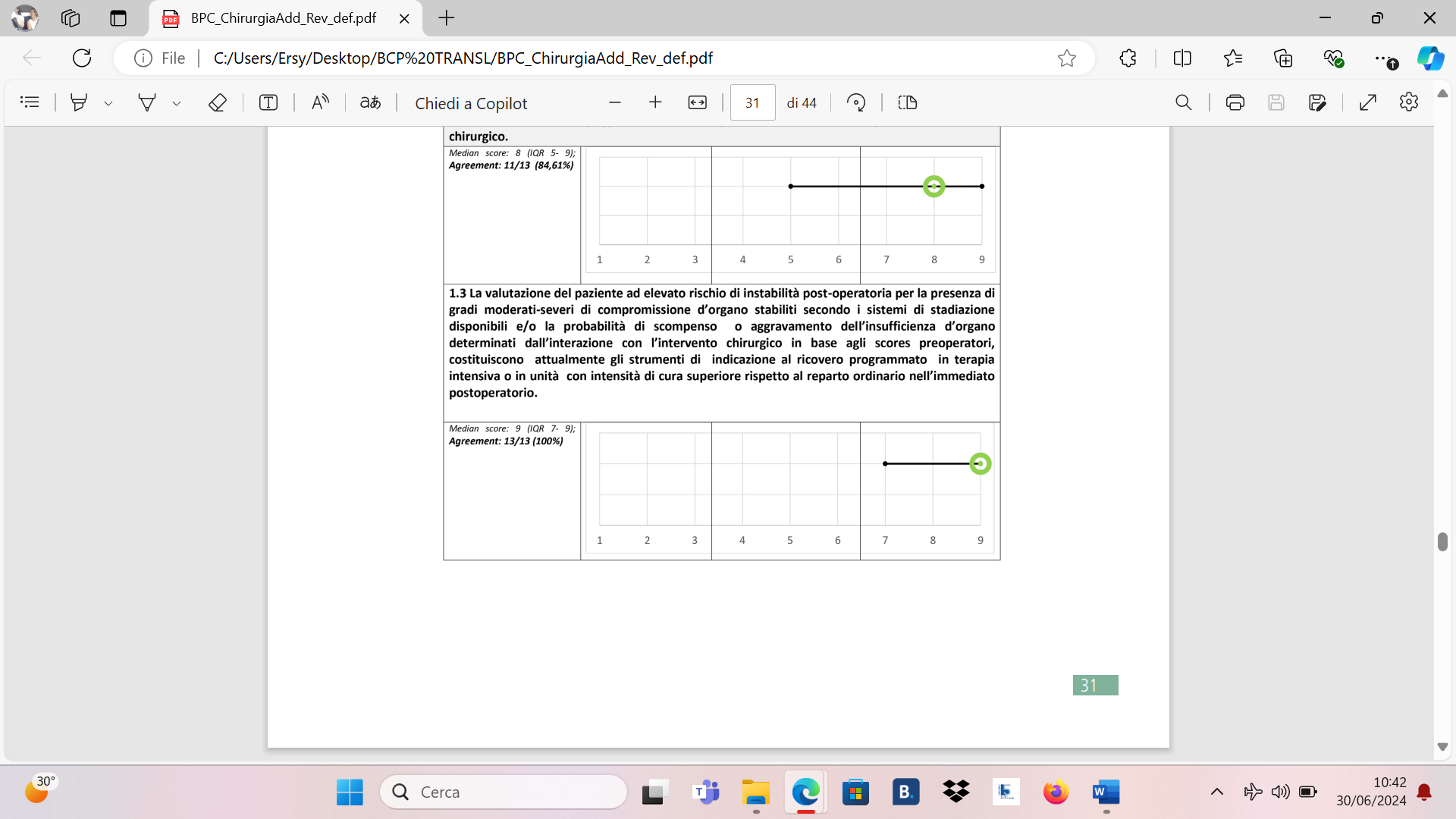 |  |  |
| **2.1 In light of current literature, the most appropriate strategy remains to plan the need for ICU admission in the presence of multiple non-modifiable pre-existing factors (e.g., ASA score** $\geq$**3, Charlson Comorbidity Index ≥2.33, Rockwood Frailty Index ≥0.25, BMI >60, male gender, diabetes, sarcopenia) and/or in the case of intraoperative events (e.g., APGAR score <7, intraoperative hemorrhage, hypotension requiring amine support, respiratory issues) that require intensive monitoring/treatment in the immediate postoperative period.** | | | |
| Median score: 9 (IQR 5-9);  **Agreement: 11/13 (84,61%)** | 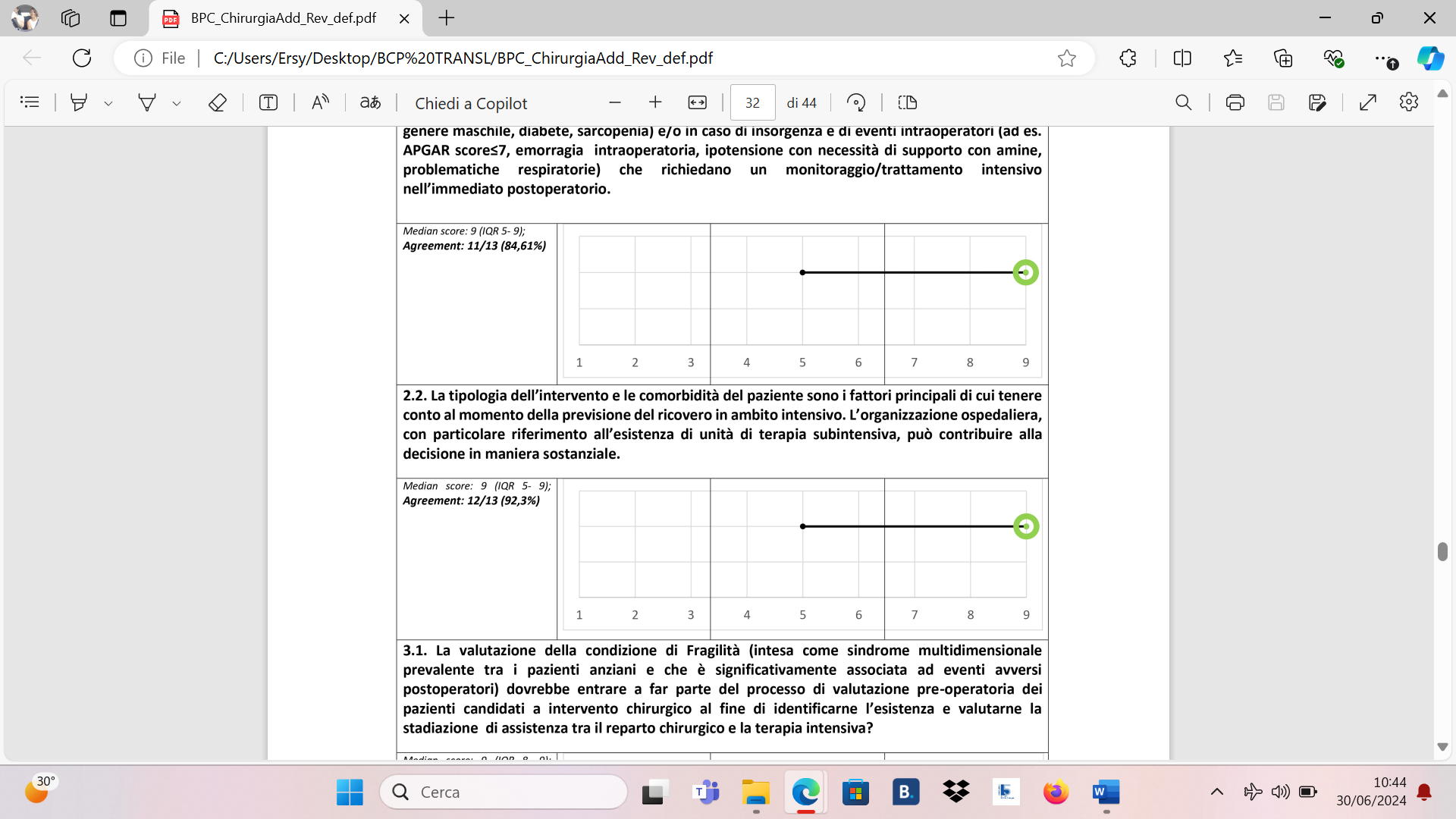 |  |  |
| **2.2 The type of surgery and the patient's comorbidities are the main factors to consider when planning intensive care admission. The hospital organization, particularly the existence of intermediate care units, can significantly contribute to this decision.** | | | |
| -Median score: 9 (IQR 5-9);  **Agreement: 12/13 (92,3%)** | 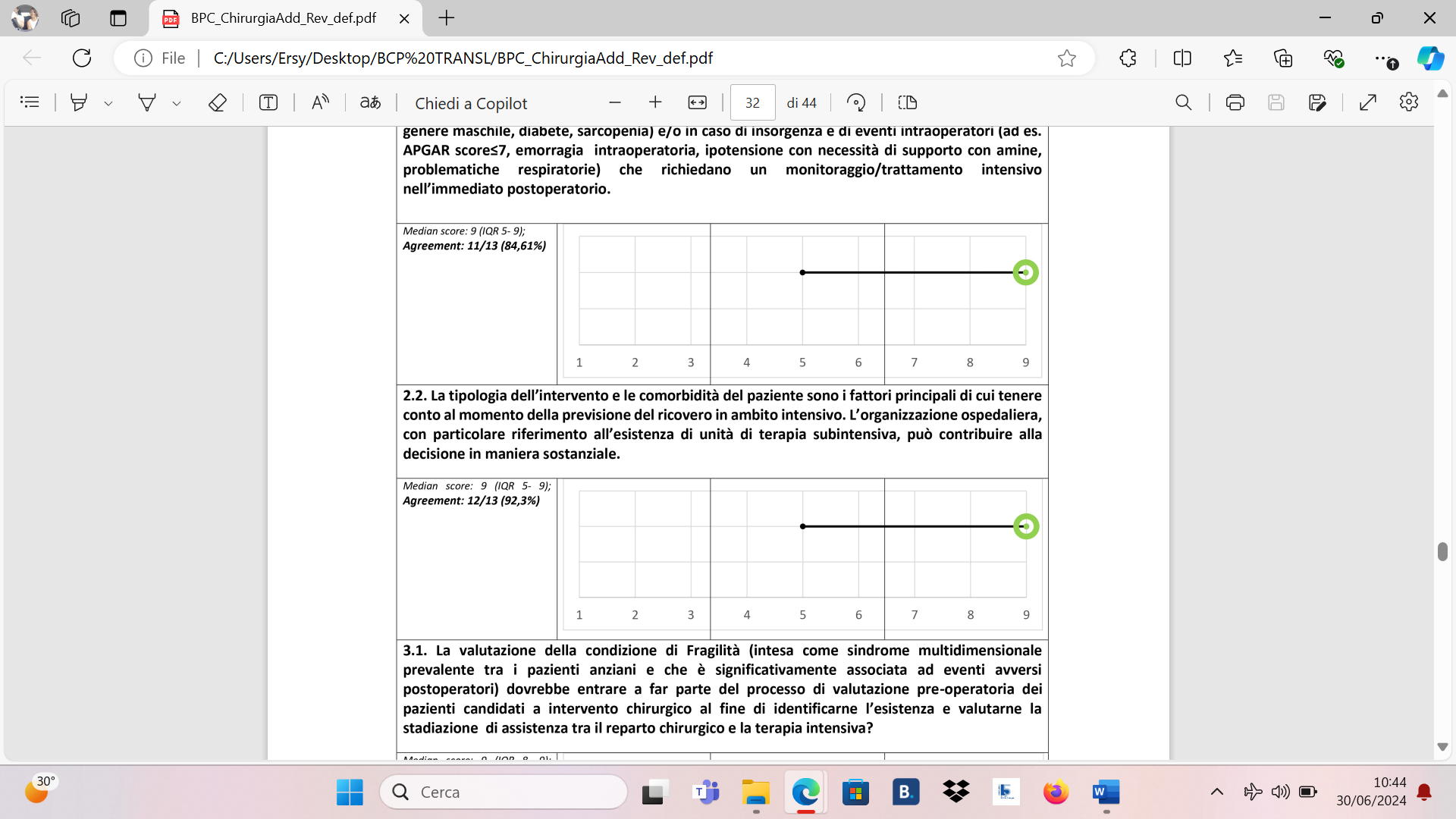 |  |  |
| **3.1 The assessment of frailty (understood as a multidimensional syndrome prevalent among elderly patients and significantly associated with postoperative adverse events) should be included in the preoperative evaluation process of patients scheduled for surgery. This is to identify its presence and evaluate the appropriate level of care between the surgical ward and intensive care.** | | | |
| Median score: 9 (IQR 6-9);  **Agreement: 13/13 (100%)** | 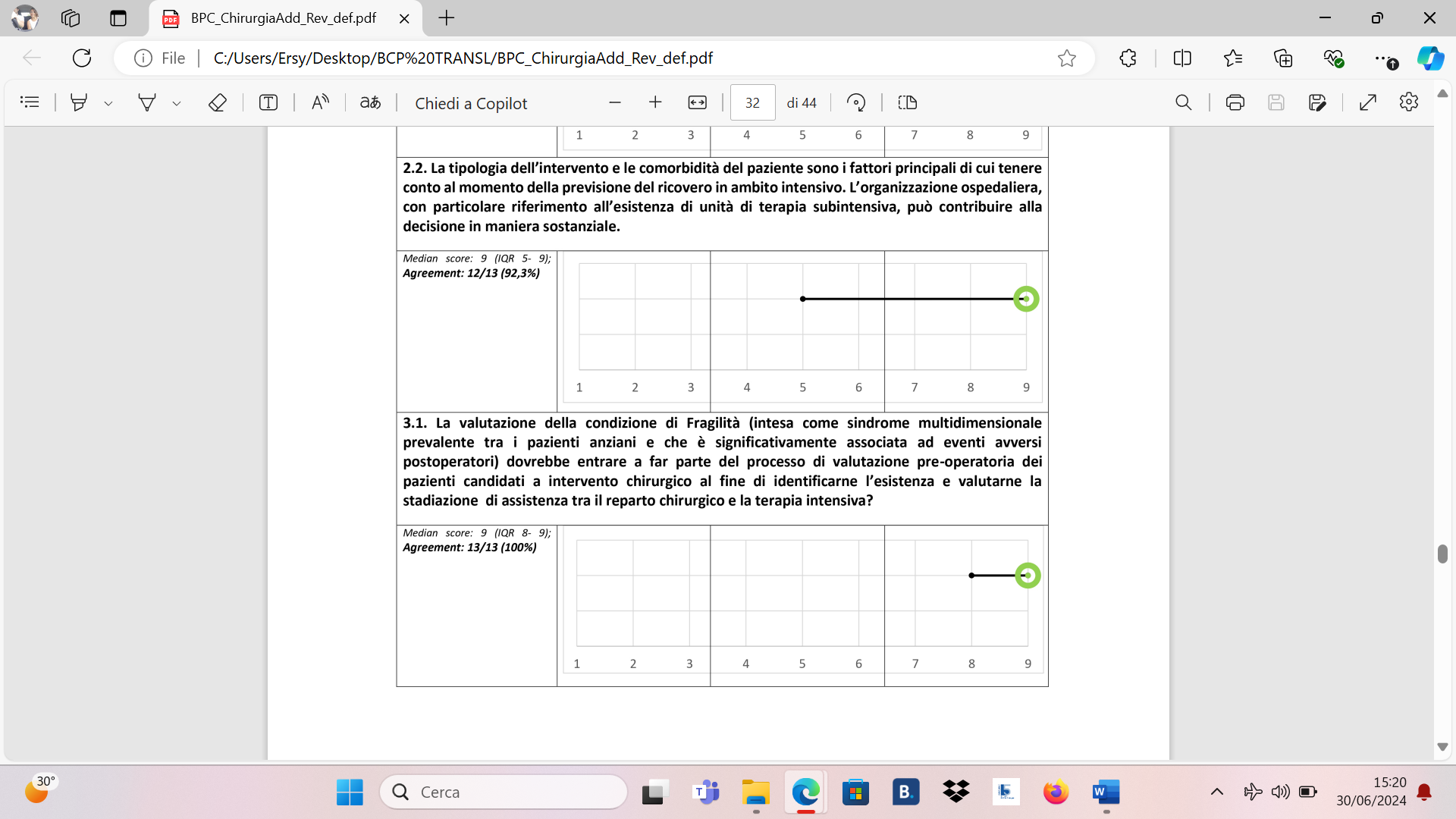 |  |  |
| **3.2 Although high-quality evidence supporting a particular frailty assessment tool over others is lacking, there is a consensus in the literature that the Clinical Frailty Scale (CFS) possesses predictive accuracy and practicality (speed and ease of use) that make it preferable for routine application in the preoperative evaluation process.** | | | |
| Median score: 9 (IQR 8-9);  **Agreement: 13/13 (100%)** | 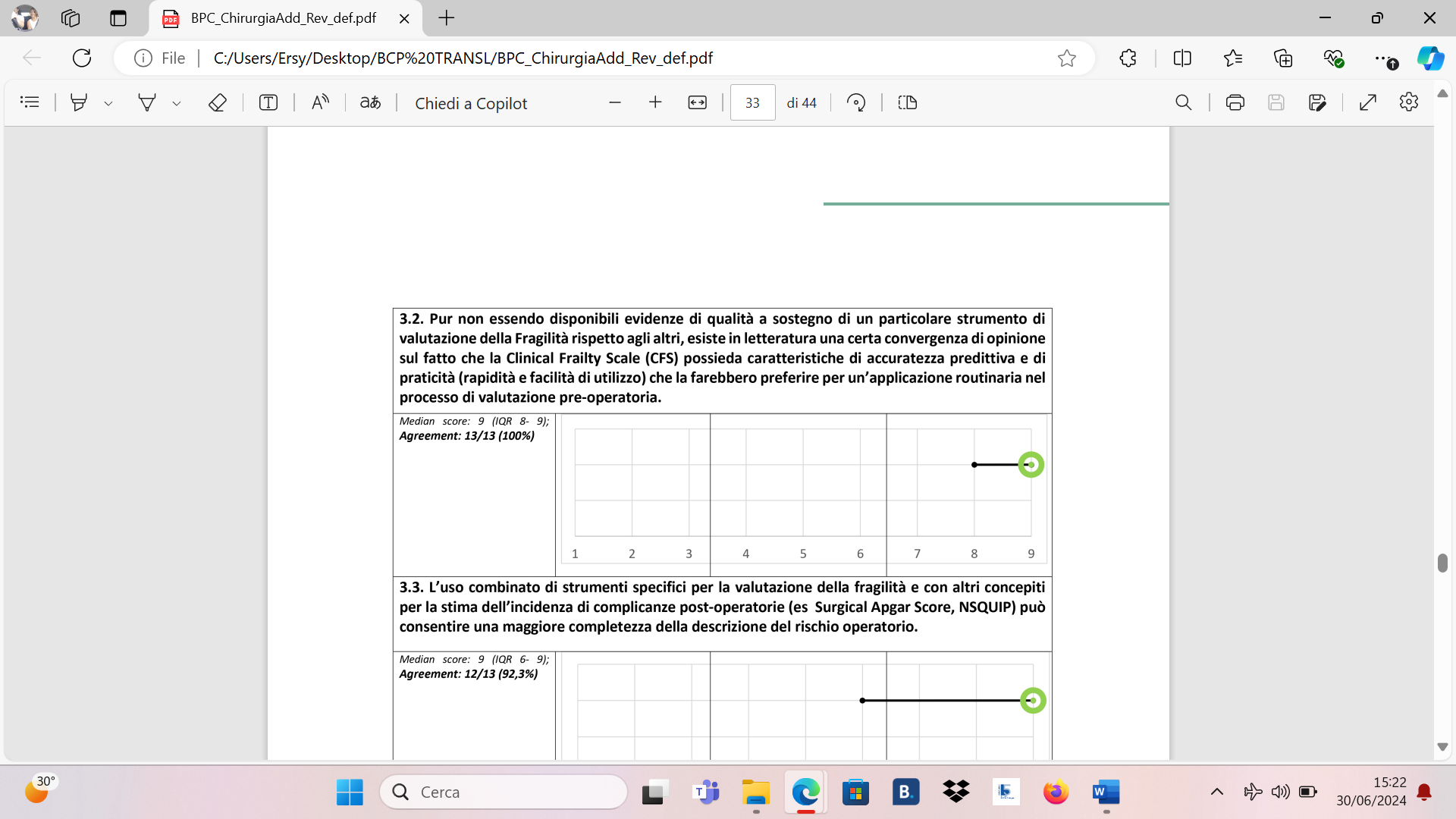 |  |  |
| **3.3 The combined use of specific frailty assessment tools along with others designed to estimate the incidence of postoperative complications (e.g., Surgical Apgar Score, NSQIP) can provide a more comprehensive description of surgical risk.** | | | |
| Median score: 9 (IQR 6-9);  **Agreement: 12/13 (92,3%)** | 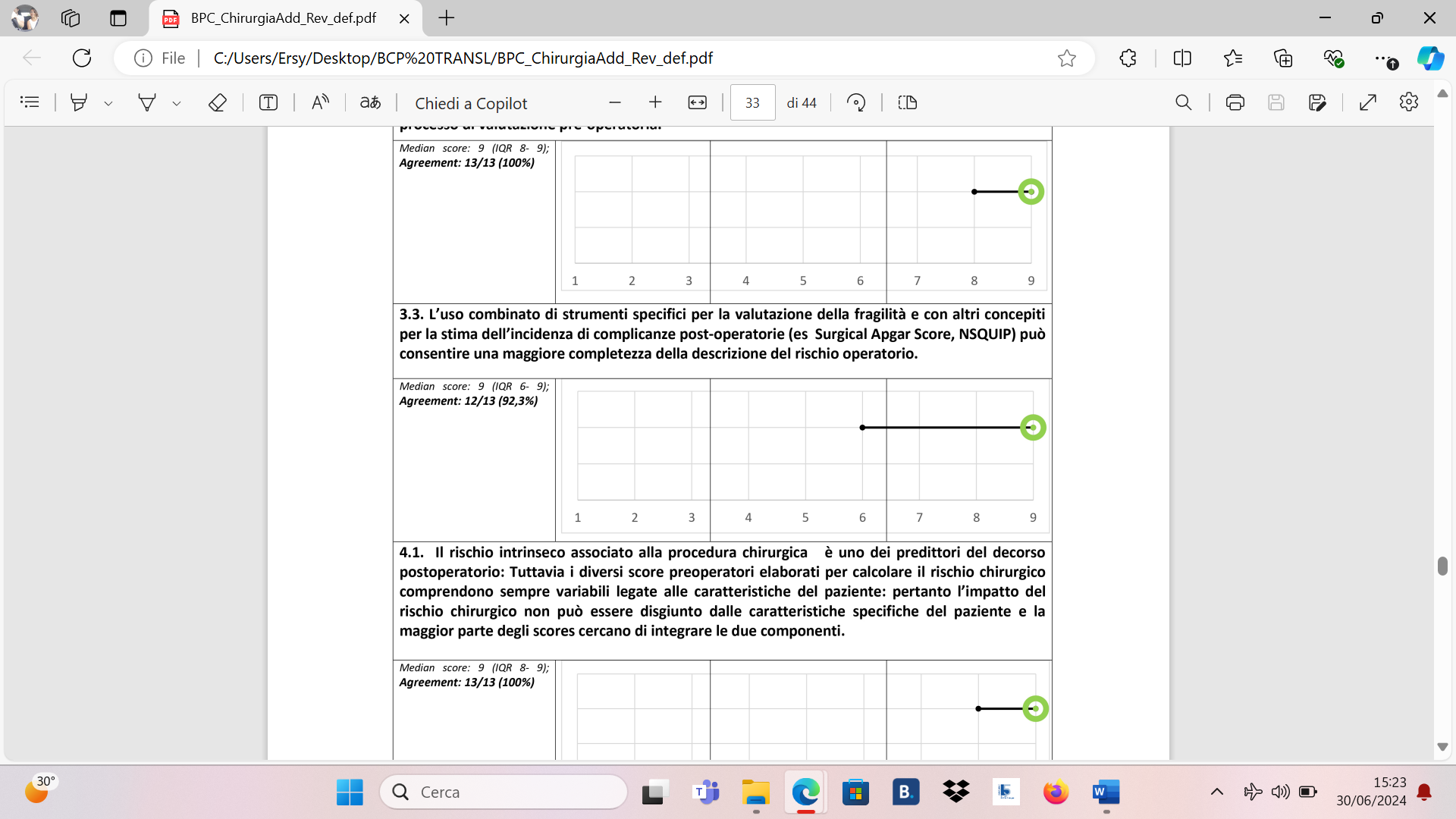 |  |  |
| **4.1 The intrinsic risk associated with the surgical procedure is one of the predictors of the postoperative course. However, the various preoperative scores developed to calculate surgical risk always include variables related to patient characteristics. Therefore, the impact of surgical risk cannot be separated from the patient's specific characteristics, and most scores aim to integrate both components.** | | | |
| Median score: 9 (IQR 8-9);  **Agreement: 13/13 (100%)** | 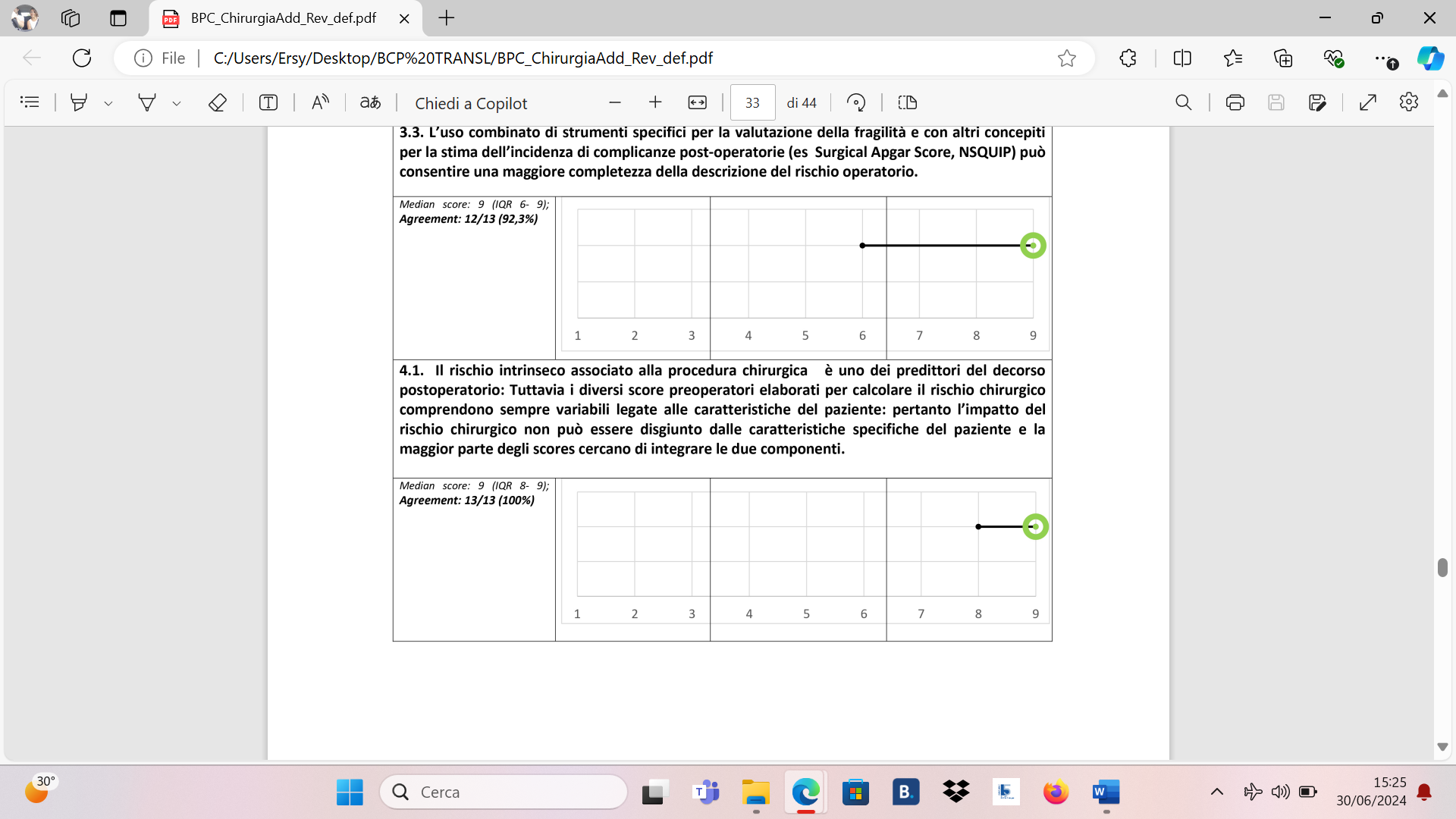 |  |  |
| **4.2 Minimally invasive surgical techniques, by reducing surgical trauma, can lower the intrinsic risk associated with the procedure and postoperative morbidity. However, ERAS (Enhanced Recovery After Surgery) programs, in which these techniques are key components, are based on multimodal perioperative interventions that optimize patient conditions (multimodal prehabilitation) and reduce the risk of surgery-induced organ dysfunction. However, these techniques can currently be applied only to selected patients based on their characteristics. The existing surgical risk scores have not been validated for surgical approaches with lower intrinsic risk.** | | | |
| Median score: 8 (IQR 1-9);  **Agreement: 10/13 (76,92%)** |  |  | 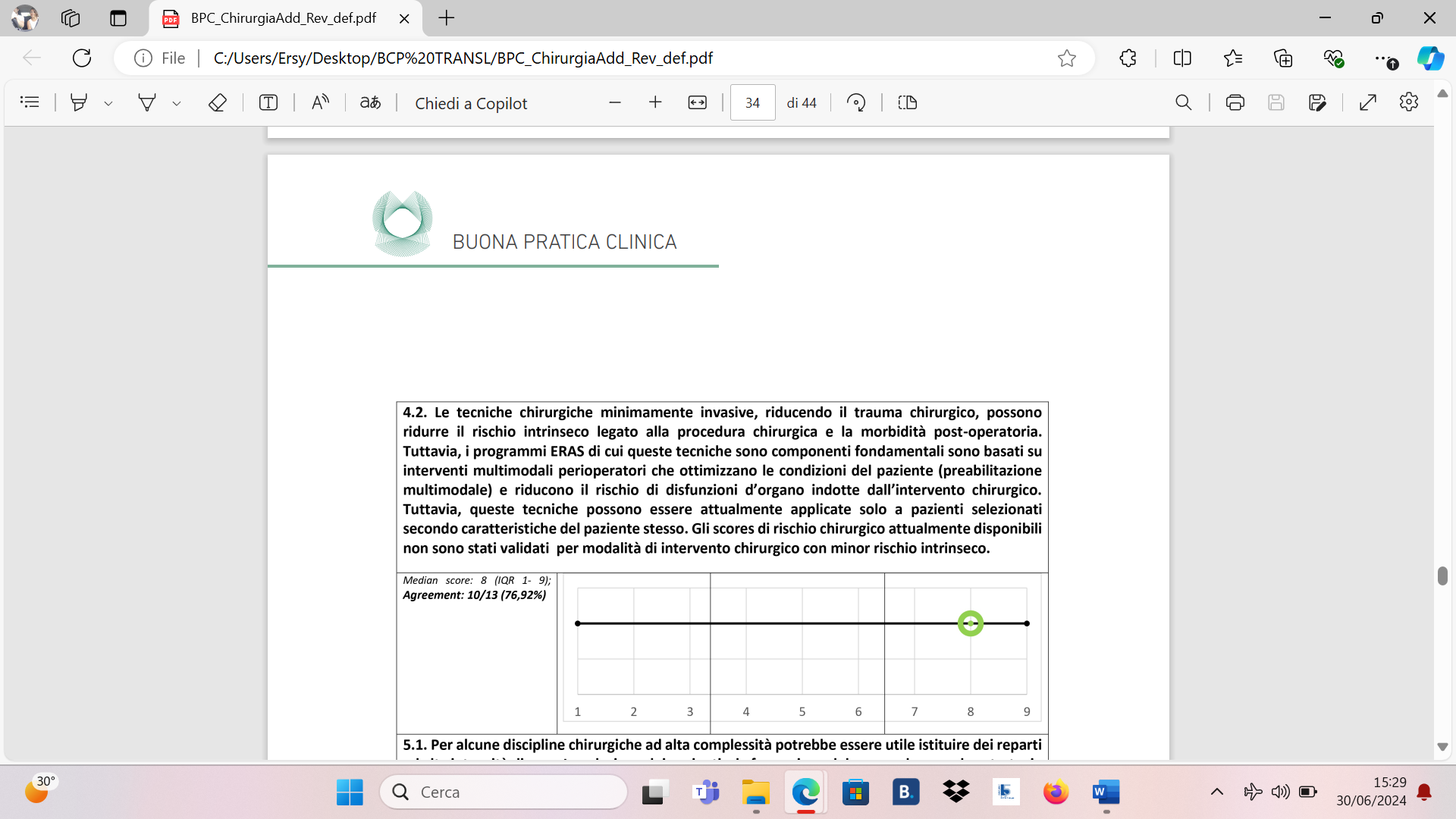 |
| **5.1 For certain high-complexity surgical disciplines, it may be beneficial to establish high-intensity care units. Patient selection and staff training are two fundamental strategies for optimizing outcomes.** | | | |
| Median score: 9 (IQR 5-9);  **Agreement: 10/13 (76,92%)** | 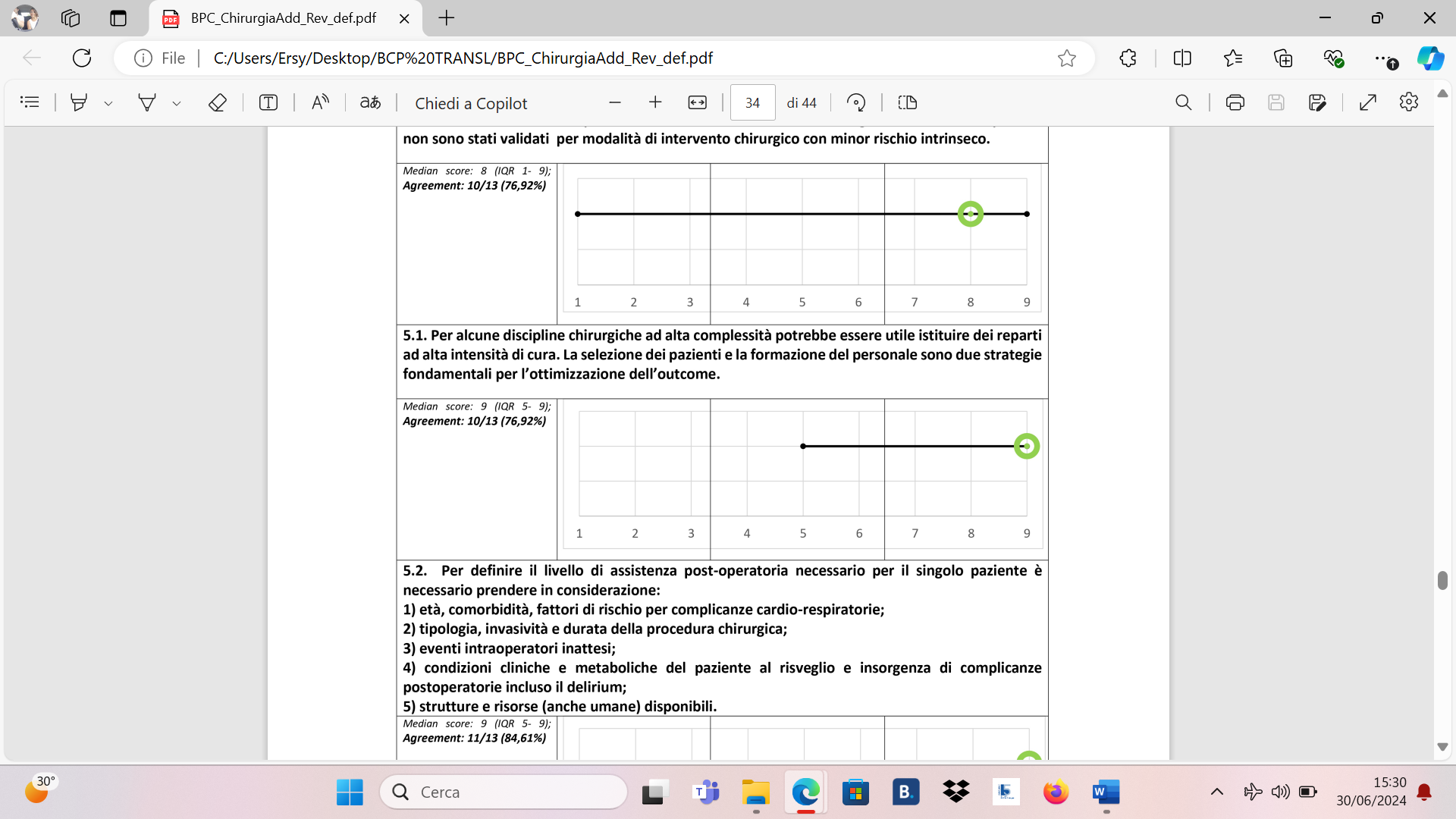 |  |  |
| **5.2 To determine the necessary level of postoperative care for an individual patient, it is essential to consider the following:**  **Age, comorbidities, and risk factors for cardiorespiratory complications;**  **Type, invasiveness, and duration of the surgical procedure;**  **Unexpected intraoperative events;**  **Clinical and metabolic condition of the patient upon awakening and the occurrence of postoperative complications, including delirium;**  **Available facilities and resources (including human resources).** | | | |
| Median score: 9 (IQR 5-9);  **Agreement: 11/13 (84,61%)** | 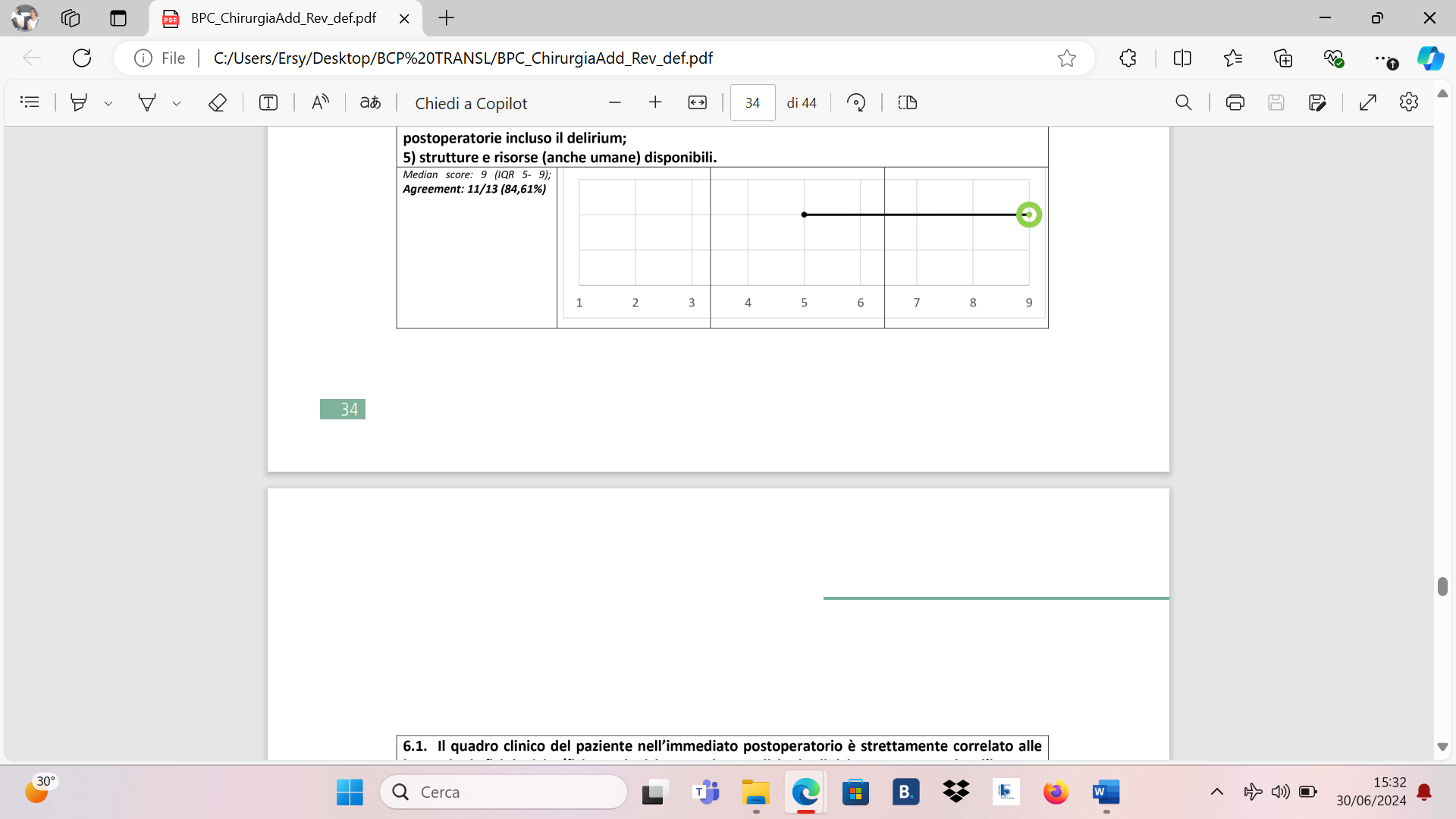 |  |  |
| **6.1 The clinical condition of the patient in the immediate postoperative period is closely related to the physiological and pathophysiological interactions between preoperative clinical conditions, the impact of the surgical procedure and the consequent stress on organs and systems, the pharmacological effects of any mechanical ventilation, and the fluids related to anesthetic management.** | | | |
| Median score: 9 (IQR 5-9);  **Agreement: 11/13 (84,61%)** |  | 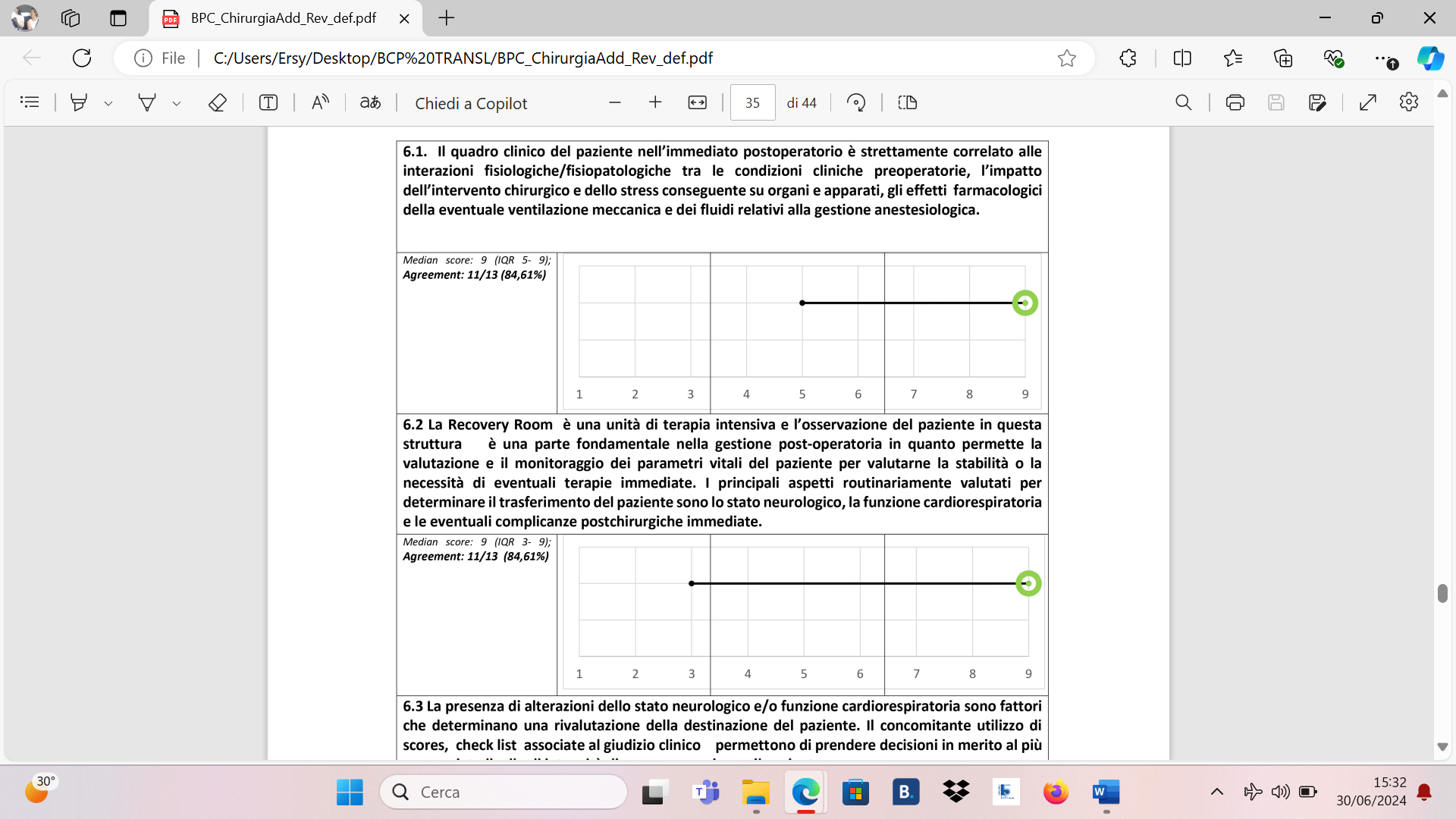 |  |
| **6.2 The Post-Anesthesia Care Unit (PACU) is an intensive care unit, and patient observation in this setting is a fundamental aspect of post-operative management. It allows for the assessment and monitoring of the patient's vital signs to determine their stability or the need for any immediate therapies. The primary aspects routinely evaluated to determine patient transfer include neurological status, cardiorespiratory function, and any immediate post-surgical complications.** | | | |
| Median score: 9 (IQR 3-9);  **Agreement: 11/13 (84,61%)** | 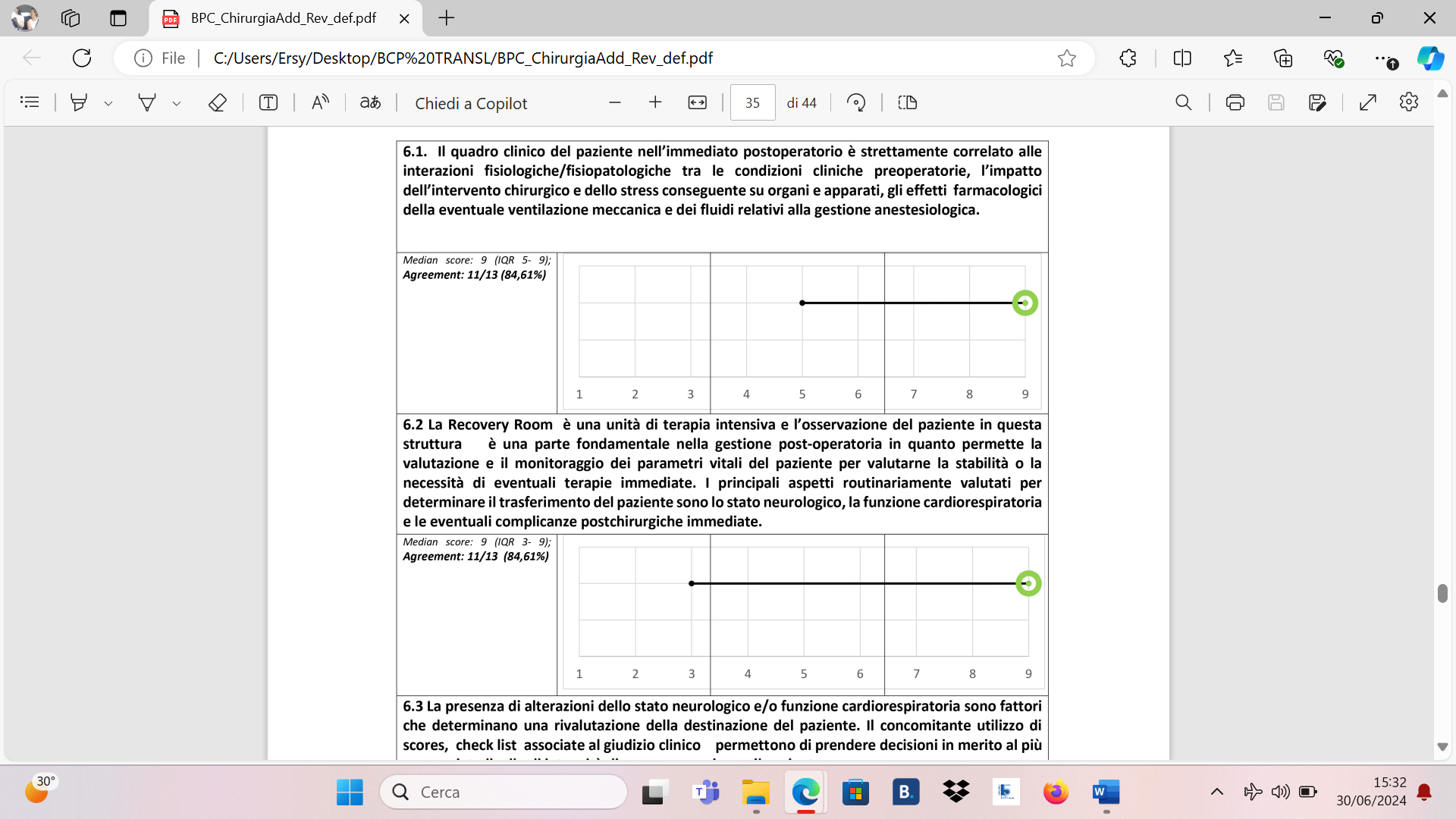 |  |  |
| **6.3 The presence of alterations in neurological status and/or cardiorespiratory function are factors that necessitate a reassessment of the patient's destination. The concurrent use of scores, checklists, and clinical judgment allows for decisions regarding the most appropriate level of intensity of care required for a single patient.** | | | |
| Median score: 9 (IQR 1-9);  **Agreement: 12/13 (92,3%)** | 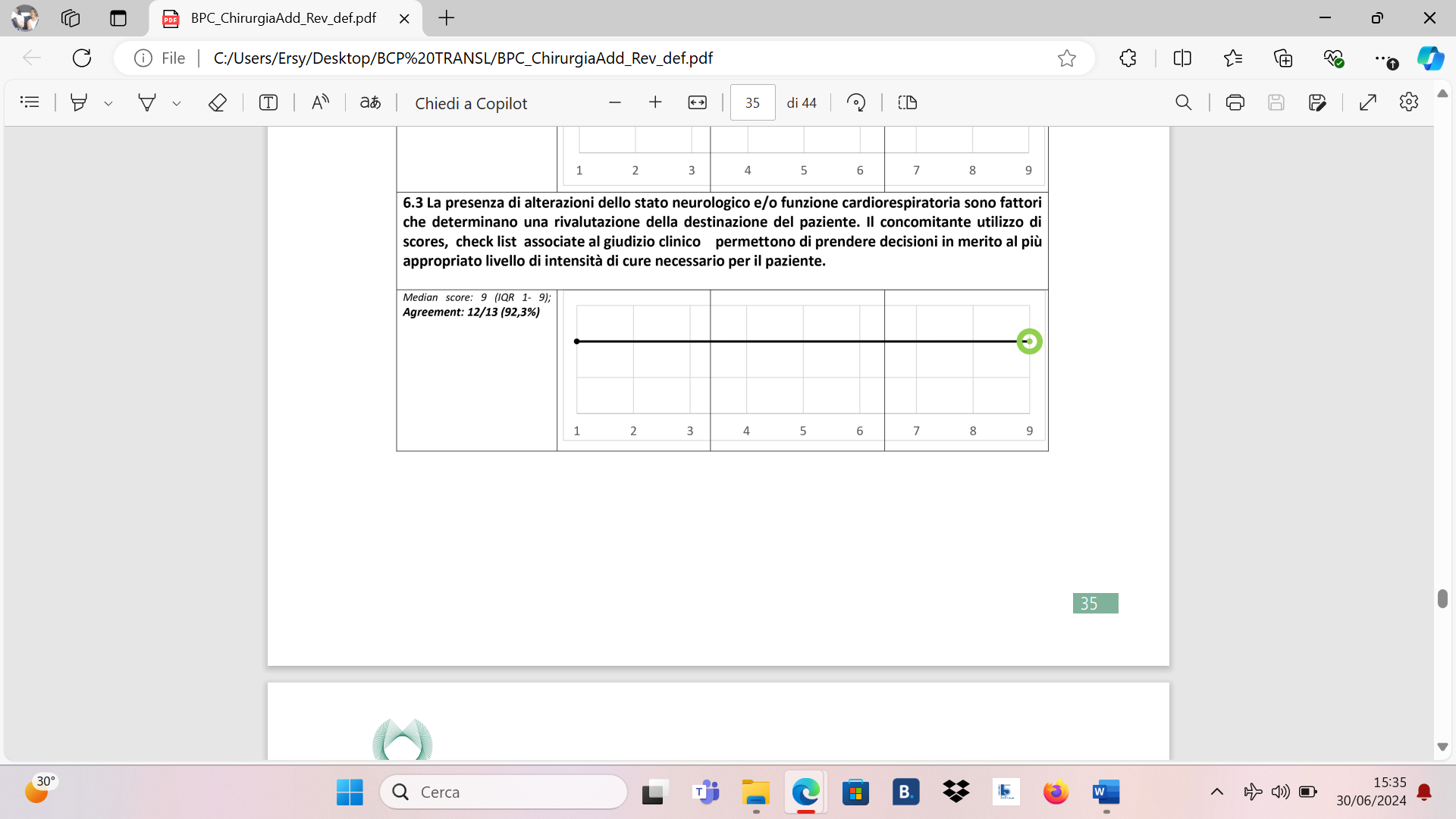 |  |  |
| **6.4 The presence of comorbidities with moderate to severe organ function impairment, even if the patient is stable, may require continuous postoperative monitoring. This should be carried out in units designated by the hospital organization where such monitoring is feasible.** | | | |
| Median score: 9 (IQR 3-9);  **Agreement: 11/13 (84,61%)** |  | 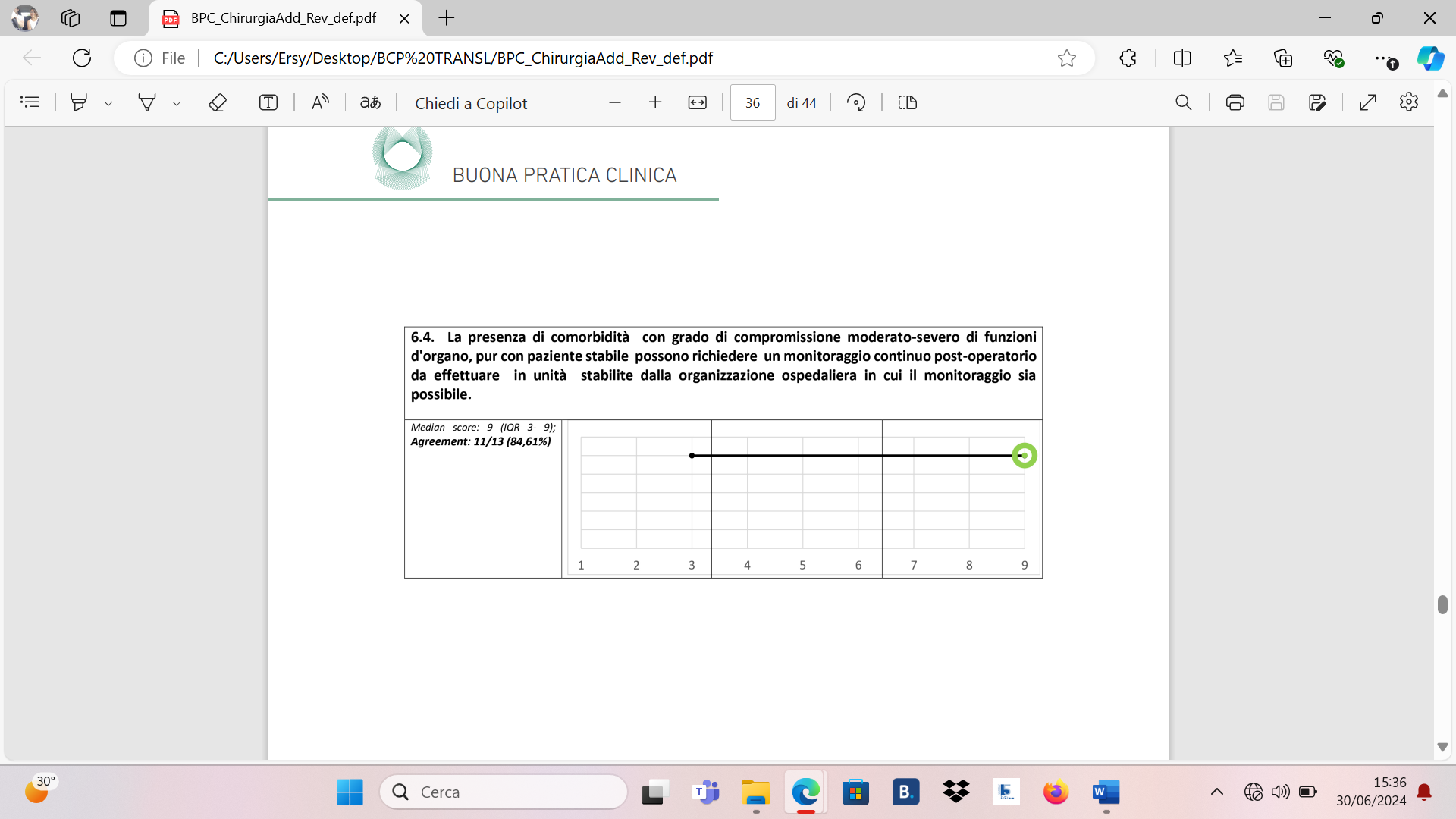 |  |

**APPENDIX 4 - PRISMA FLOW**

**CQ1 - What are the patient characteristics/comorbidities that define, in an objective and systematic manner, the appropriateness for postoperative admission to the intensive care unit?**
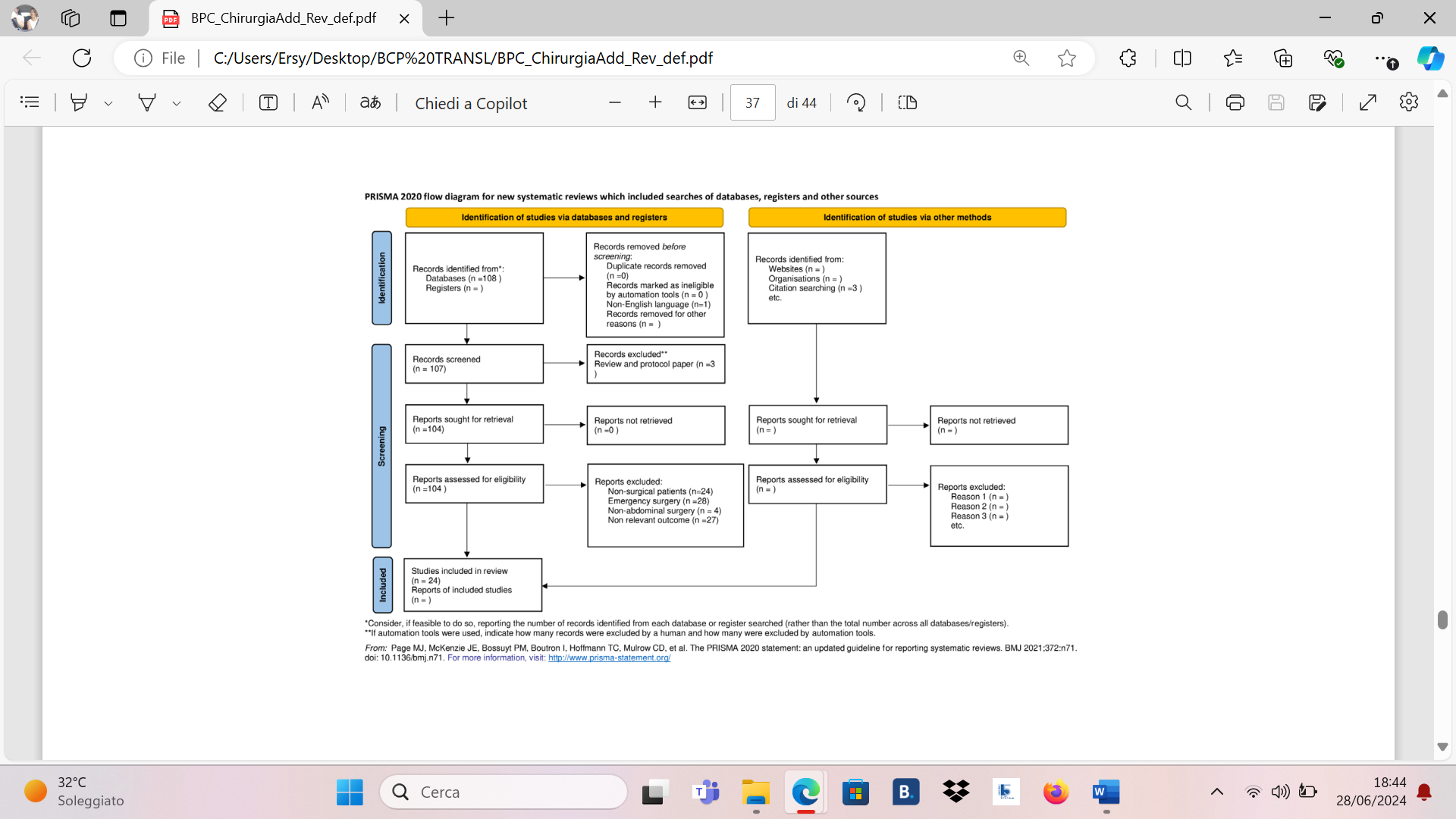


CQ2 – **What are the appropriate tools to objectively and systematically define the indication for postoperative admission to the intensive care unit?**

**
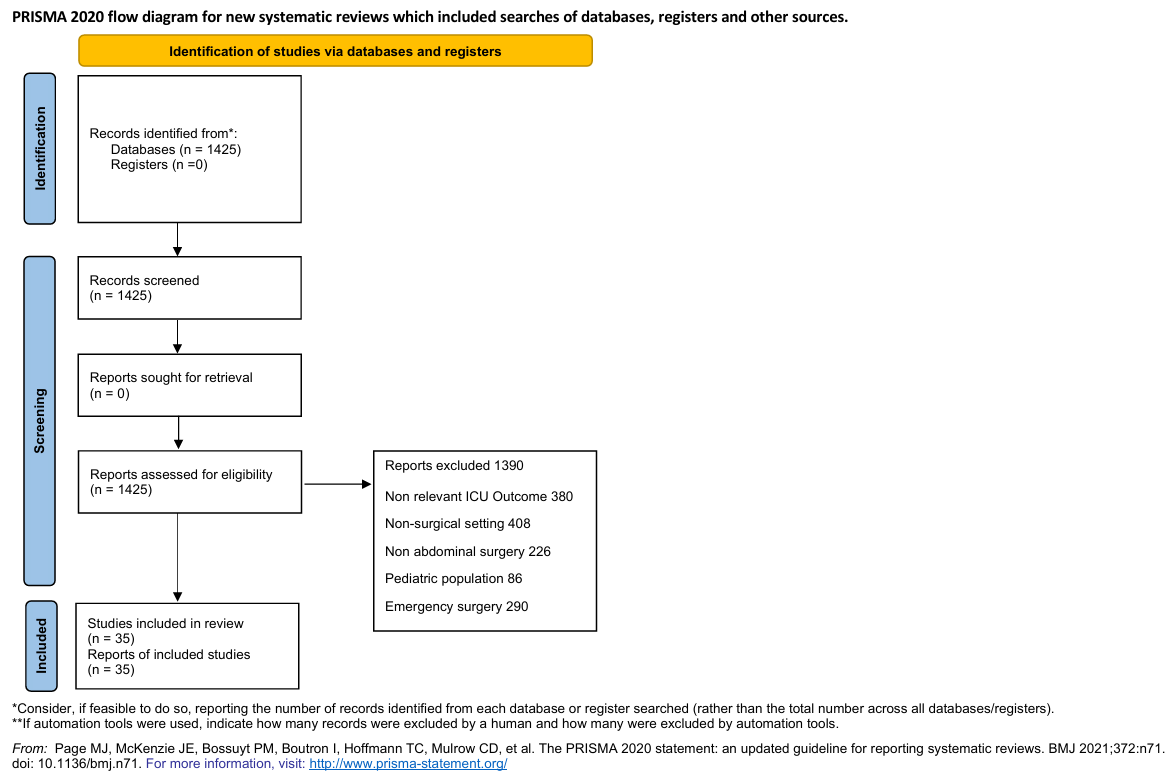
**

**CQ3 – What are the appropriate tools to assess the degree of frailty preoperatively in patients to determine the indication for postoperative admission to the intensive care unit?**

**
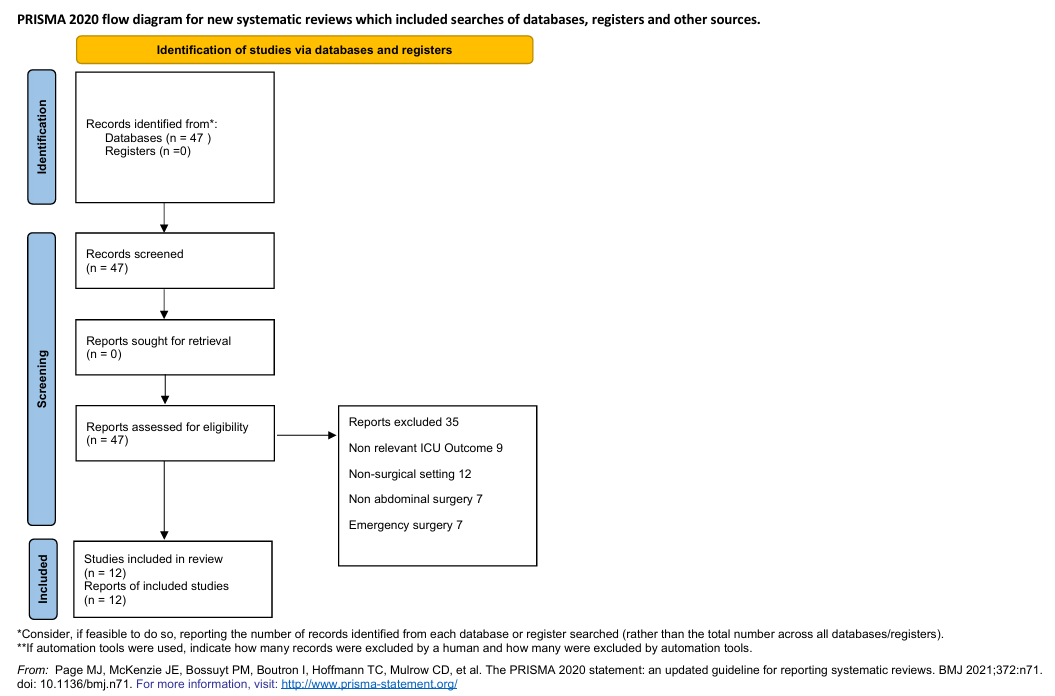
**

**CQ4 – Which tools allow for assessing the impact of surgical intervention and/or surgical technique on the physiology of individual patients to determine the need for postoperative admission?**


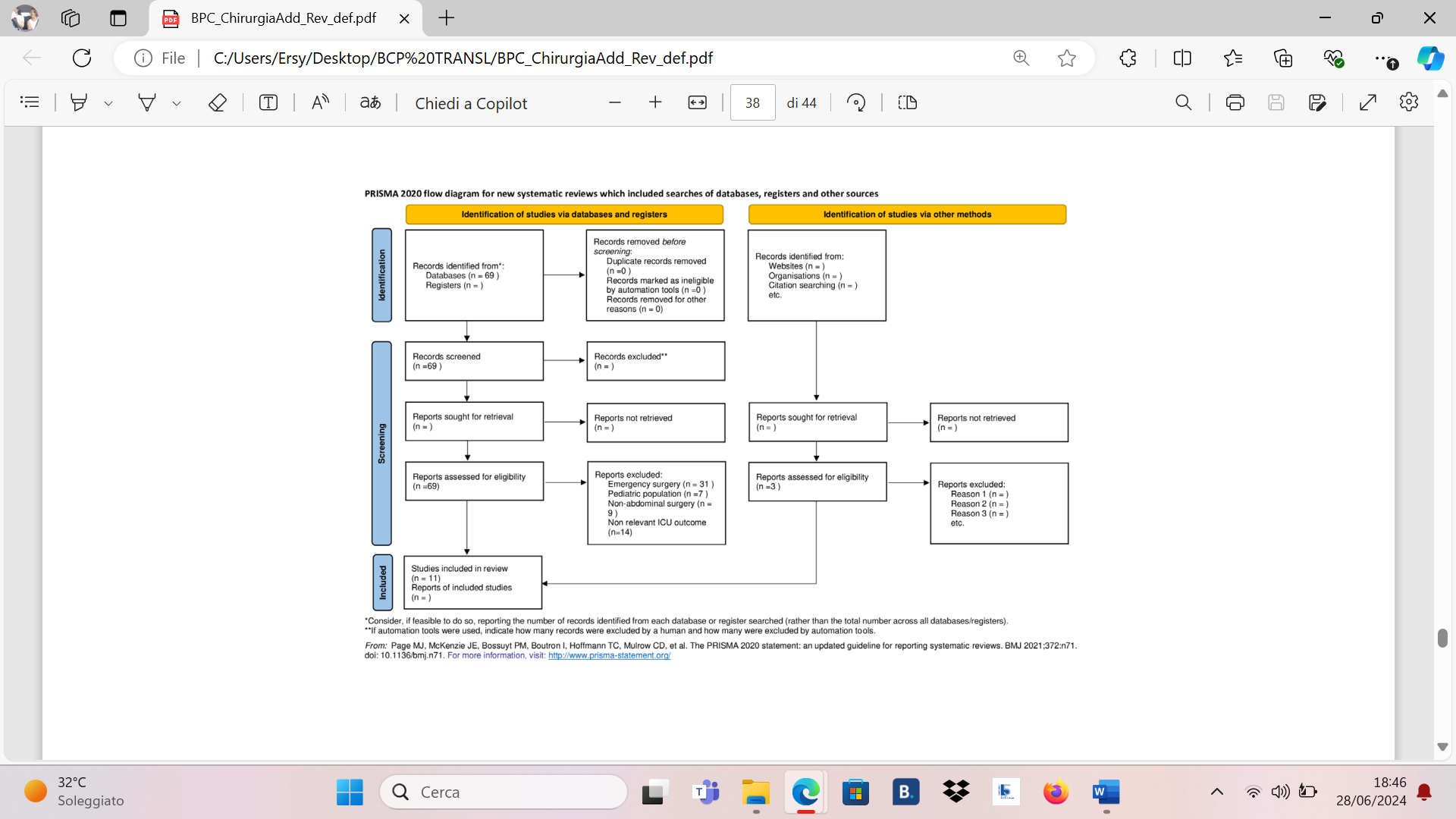


**CQ5 – Identifying criteria and profiles for postoperative monitoring and support can define intermediate levels of care between the surgical ward and intensive care unit (ICU).**

**
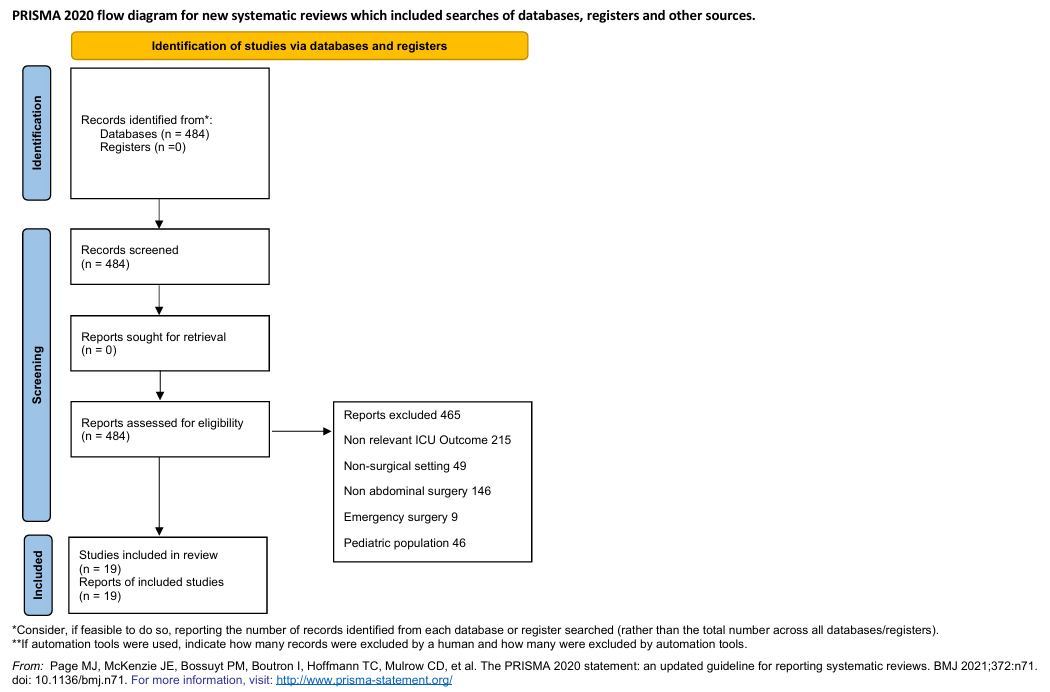
**

**CQ6- Which parameters should be reassessed at the end of the procedure and before discharge from the recovery room to confirm, indicate, or modify the indication for intensive care unit (ICU) admission?**
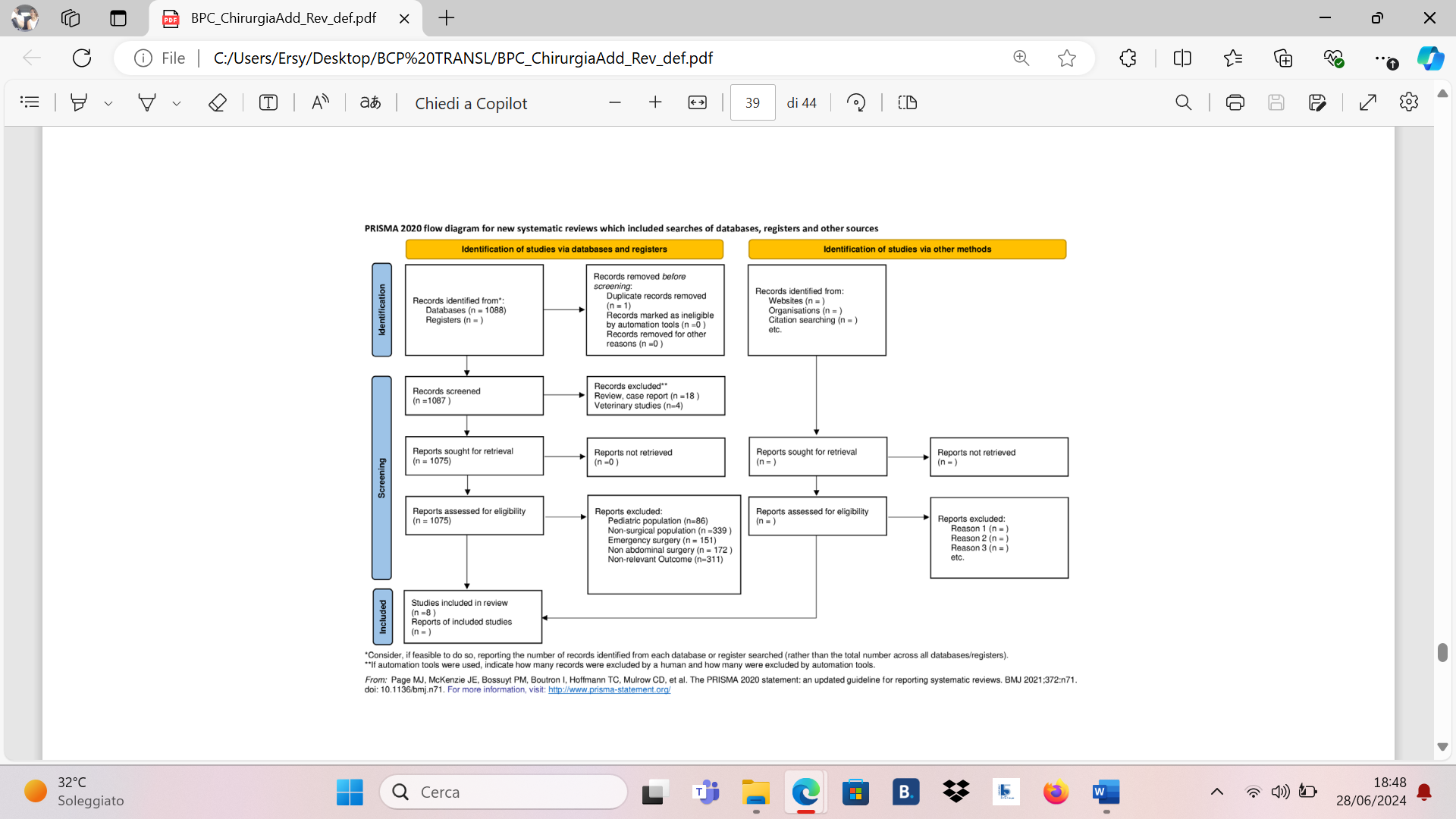


**APPENDIX 5 PROPOSAL OF DECISIONAL ALGORITHM**

Proposal of decisional algorithm for the perioperative pathway of patients undergoing high-complexity abdominal surgery


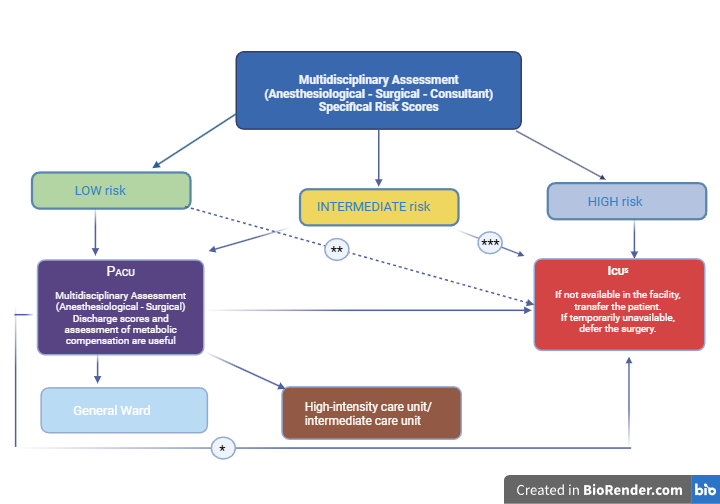


*If not available in the facility, transfer to Intensive Care Unit

**Consider intraoperative events (e.g., Surgical APGAR score ≤7, intraoperative hemorrhage, hypotension requiring vasopressor support, respiratory issues).

***Consider the combination of multiple pre-existing non-modifiable factors (e.g., ASA score ≥3, Charlson Comorbidity Index ≥2, Rockwood Frailty Index ≥0.25, Revised Cardiac Risk Index (Lee Criteria) ≥2, BMI >60, male gender, diabetes, sarcopenia) and/or intraoperative events (e.g., Surgical APGAR score ≤7, intraoperative hemorrhage, hypotension requiring vasopressor support, respiratory issues).

§ Useful multidisciplinary definition of criteria for transfer from Intensive Care Unit to lower intensity care unit/general ward
